# Characterization of the Coordination and Solvation Dynamics of Solvated Systems—Implications for the Analysis of Molecular Interactions in Solutions and Pure H_2_O

**DOI:** 10.1021/acs.jctc.4c00162

**Published:** 2024-04-10

**Authors:** Risnita Vicky Listyarini, Bernhard M. Kriesche, Thomas S. Hofer

**Affiliations:** † Institute of General, Inorganic and Theoretical Chemistry Center for Chemistry and Biomedicine, University of Innsbruck Innrain 80-82, A-6020 Innsbruck, Austria; ‡ Chemistry Education Study Program Sanata Dharma University, Yogyakarta 55282, Indonesia

## Abstract

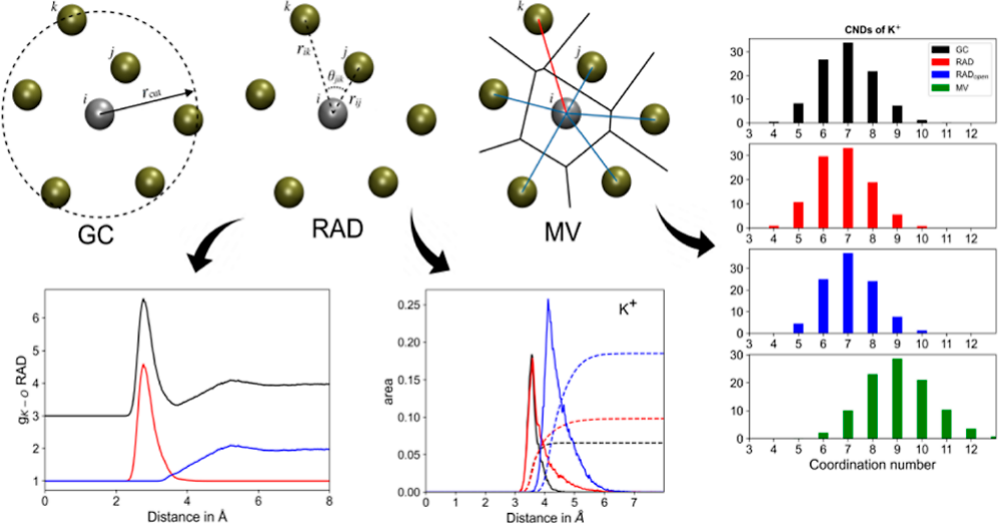

The characterization of solvation shells of atoms, ions, and molecules in solution is essential to relate solvation properties to chemical phenomena such as complex formation and reactivity. Different definitions of the first-shell coordination sphere from simulation data can lead to potentially conflicting data on the structural properties and associated ligand exchange dynamics. The definition of a solvation shell is typically based on a given threshold distance determined from the respective solute–solvent pair distribution function *g*(*r*) (i.e., GC). Alternatively, a nearest neighbor (NN) assignment based on geometric properties of the coordination complex without the need for a predetermined cutoff criterion, such as the relative angular distance (RAD) or the modified Voronoi (MV) tessellation, can be applied. In this study, the effect of different NN algorithms on the coordination number and ligand exchange dynamics evaluated for a series of monatomic ions in aqueous solution, carbon dioxide in aqueous and dichloromethane solutions, and pure liquid water has been investigated. In the case of the monatomic ions, the RAD approach is superior in achieving a well separated definition of the first solvation layer. In contrast, the MV algorithm provides a better separation of the NNs from a molecular point of view, leading to better results in the case of solvated CO_2_. When analyzing the coordination environment in pure water, the cutoff-based GC framework was found to be the most reliable approach. By comparison of the number of ligand exchange reactions and the associated mean ligand residence times (MRTs) with the properties of the coordination number autocorrelation functions, it is shown that although the average coordination numbers are sensitive to the different definitions of the first solvation shell, highly consistent estimates for the associated MRT of the solvated system are obtained in the majority of cases.

## 1 Introduction

The significance of solvation in the reactivity of chemical species such as atoms, ions, and molecules in solution is widely recognized.^1–4^ The exchange of solvent molecules from the primary solvation layer can be considered as an initial step in enabling chemical reactions and complex formation.^5,6^ The different properties of solvation structures and the associated kinetics of solvent exchange have been extensively investigated by using both experimental as well as theoretical approaches. Experimentally, techniques such as X-ray/neutron diffraction and scattering^7–11^ as well as extended X-ray absorption fine structure and X-ray absorption near edge structure spectroscopy^12–14^ are typically employed to study the coordination of dissolved ions and molecules by solvent molecules. To characterize the dynamics of solvent exchange, nuclear magnetic resonance^5,10^ and incoherent quasi-elastic neutron scattering^15–19^ have been employed. Typically, experimental studies require sufficiently concentrated solutions of the dissolved compounds, which makes an extrapolation of the measured data toward low concentration and infinite dilution difficult. In addition, the methodical limit in the time resolution is typically given on the order of 10^–9^ s, i.e., on the nanosecond scale. Ultrafast processes occurring on the picosecond times scale as reported for instance in case of the first shell mean ligand residence times (MRTs) of the monovalent ions Rb^+^^20^ and Cs^+^^21^ are, in general, not accessible via these methods. Ultrafast spectroscopy^22–24^ at the femto- and even attosecond scale has been repeatedly demonstrated of being capable to provide data of ligand exchange dynamics in solution and an increasing number of investigations focused on solvent exchange dynamics of small molecular and ionic compounds are reported in the literature.^25–27^

**Figure 1:**
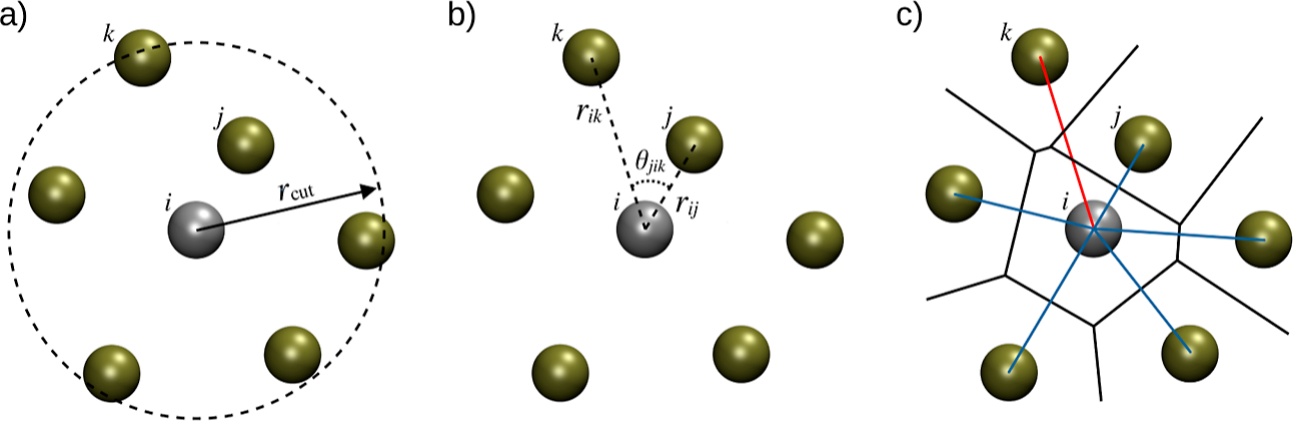
Overview of ligand assignment strategies to the first coordination sphere according to a (a) radial cutoff criterion determined from the respective RDF *g*(*r*) (GC), the (b) RAD algorithm, and the (c) modified Voronoi approach. Depending on the applied method, ligand *k* is either included or excluded from the first coordination sphere.

In contrast to experimental approaches, theoretical investigations, notably molecular dynamics (MD) simulations,^28^ are perfectly capable of studying solutions at very low concentrations. In the most extreme case, only a single dissolved species can be investigated, which effectively corresponds to infinite dilution. By employing appropriate approaches to the treatment of the long-ranged Coulombic interactions, this concept can even be extended toward the treatment of a single ionic solute in solution.^29–32^ In particular, when a quantum chemical description of the interactions is used, such as in Car–Parrinello (CP)^33^ and hybrid quantum mechanical/molecular mechanical^34,35^ (QM/MM) MD simulations, data in very good agreement with experimental references can be obtained. Since MD simulations are typically executed in time steps at the (sub)femto-second range, these approaches are perfectly suited to study ultrafast ligand exchange dynamics occurring on the picosecond scale.^36–39^ However, apart from the challenges associated with the accurate description of molecular interactions, the definition of a solvation shell may in some cases be ambiguous. In case of simple solutes such as solvated, monatomic ions, a simple radius-dependent criterion based on solute–solvent pair distribution functions (i.e., radial distribution functions *g*(*r*), RDFs) as shown in Figure 1a is the default method to characterize individual solvation shells.^40–42^ Since the oxygen atom in a H_2_O molecule is in close vicinity to its center of mass, it is oftentimes sufficient to identify the minimum in between the first and second shell peaks of the respective ion-O RDF. However, already in the case of solvated anions, this approach can be shown to result in ambiguous data. In a recent QM/MM MD study^43^ of aqueous F^–^, Cl^–^, and Br^–^, the application of a cutoff criterion based on the respective RDFs *g*(*r*) resulted in coordination numbers (CNs) showing notable deviations from experimental estimates, while other structural properties such as the average first shell ion-oxygen distance were found in good agreement with experimental and theoretical reference data. In order to improve the determination of the CN in case of these more complex hydrogen-bonded complexes, an alternative definition of the first shell neighbors based on the relative angular distance (RAD) algorithm introduced by Higham and Henchman^44^ was employed. This framework is based on the geometric concept of the solid angle Ω, which quantifies the field of view of a given object, e.g., an atom of a solvent molecule, from a predefined reference point, which, in this case, represents the solute. In order to determine which solvent molecule is considered as part of the first solvation layer, the solute–solvent distances are evaluated based on a combined, angular-radial criterion (cf. Figure 1b). In the RAD method, solvent molecules must be first sorted by distance from the solute *i* in ascending order. In the next step, a molecule *j* is considered as a member of the initial solvation layer if it is unblocked by all previously identified closer ligands *k*, according to the criterion
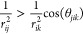
1with *r*_*ij*_, *r*_*ik*_, and θ_*jik*_ being the associated ion–ligand pair distances and the solvent–solute–solvent angle, respectively. In the default implementation of the RAD method, the incremental search for ligands in the solvation shell is ended as soon as the first ligand *j*′ violates the RAD criterion and is considered as blocked. Alternatively, a less-tight criterion referred to as RAD_open_ has been introduced, in which the search is continued even though closer ligands are identified as blocked.^44^ A key advantage of the RAD algorithm is the classification of first shell ligands without the requirement of any predetermined cutoff criterion solely on the basis of geometric properties of the coordination complex. By applying this alternative characterization of first shell ligands, a much better agreement in the CN with experimental reference data could be achieved when analyzing F^–^, Cl^–^, and Br^–^ in aqueous solution^43^ as well as when characterizing pure liquid systems.^45^

Another method similar to the RAD analysis is the modified Voronoi (MV) method developed by Malins et al.^46^ In this framework, two additional criteria to the standard Voronoi analysis^47^ are introduced since it is known that the latter tends to overestimate the number of nearest neighbors (NNs).^45,46,48^ First, a particular atom *j* is only considered as a “direct neighbor” of solute *i* if the shared face of the respective Voronoi cells is intersected by its corresponding pair vector **r**_*ij*_ (cf. Figure 1c). All neighbor atoms adhering to this criterion are termed “direct neighbors” and are considered as part of the first solvation shell. Similar to the case of the RAD analysis, a simple inequality based on the pair distances *r* of all involved species *i*, *j*, and *k* can be provided
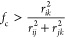
2with the parameter *f*_c_ balancing the asymmetry in the detection of four-membered rings versus a classification considering four particles as being arranged in two separate three-membered rings.^46^ In case *f*_c_ is set to 1, the definition of the direct Voronoi neighbors as discussed above is exactly applied, while lower values in *f*_c_ loosens the criterion to accept additional ligands as NNs. While appearing similar in the classification of the coordination shell, a notable difference between the MV and RAD algorithms is the fact that the latter is asymmetric; i.e., two particles may disagree whether they are included in each other’s coordination sphere.

The possibility to define the first shell coordination without any predefined cutoff criterion also provides new ways in determining the associated MRTs τ. In this work, the RAD and MV approaches are combined with the highly successful direct evaluation of ligand exchange.^37^ The latter was introduced as an alternative to the exponential decay method of Impey et al.,^49^ which has been proposed as a method to determine MRT values while avoiding the necessity for any elaborate book keeping. In contrast, the direct method monitors all migrations of ligands to/from a particular solvation shell.^37^ Similarly, to the definition of the first shell coordination sphere, the original formulations of both the Impey as well as the direct method may suffer from an ill-defined definition of NNs as both formalisms were initially based on a simple radial cutoff criterion to define a particular solvation layer. However, it is straightforward to adapt the direct method to employ the above-mentioned unbiased RAD and MV approaches to identify and update the NN ligands over the course of a simulation trajectory.

Technically, the determination of NNs and the analysis of the associated ligand exchange dynamics proposed in this work are two-step procedures. First, the simulation trajectory is probed configuration by configuration to identify the time evolution of the first shell ligands based on the respective NN algorithm. After the assignment of ligands to the respective regions, the MRT analysis can be performed by independently monitoring the exchange dynamics of every solvent molecule in question. Although it is straightforward to combine these two analysis procedures into a single program, the steps have been carried out in succession to also analyze the performance of the NN algorithms in terms of the structural decomposition and the associated CN autocorrelations. While in case of monovalent cations no discernible difference to a radial cutoff criterion is expected,^41,42^ it remains to be seen to what extent ligand exchange dynamics are influenced when investigating monatomic anions in solution.^43^ A more challenging target combining cationic and anionic interaction motifs is the solvated CO_2_ molecule, thereby considering both aqueous^50^ as well as dichloromethane (DCM) solutions.^51^ As the final example, the ligand exchange dynamics of pure water is investigated by considering a preselected H_2_O molecule as solute that is solvated by all remaining molecules in the simulation cell. This analysis is then repeated for each water molecule in the solvation cell, providing an estimate of the consistency of the approaches used.

## 2 Methods

### 2.1 Structural Analysis and Characterization of Exchange Dynamics

The determination of ligand exchange dynamics was carried out using a direct evaluation approach.^49^ Typically, the assignment of ligands to a particular solvation shell is based on a simple, radial cutoff criterion (referred to in the following as GC^44^), with the exact distances being determined based on the respective ion–solvent RDF *g*(*r*). However, the classification can be easily modified to consider an alternative assignment approach, which in this study are the RAD, RAD_open_, and MV algorithms, discussed above. In the latter case, only the variant based on an *f*_c_ value of 1.0 was considered since smaller values in *f*_c_ may result in the inclusion of molecules that are not defined as “direct ligands”.

Irrespective of the method applied to assign ligands to the respective solvation shell, the determination of the associated exchange dynamics follows the same procedure. Whenever a ligand enters or leaves a coordination sphere of interest, its trajectory is followed. In case this exchange persists for more than the minimum excursion time t*, it is considered as a valid exchange event. Impey and co-workers recommended a value of 2.0 ps for t*^49^ based on an MRT value of 1.8 ps obtained for pure water at 286 K using the Matsuoka, Clementi and Yoshimine water model.^52^ Later, a detailed analysis of the QM/MM MD simulation trajectories of pure water as well as 14 mono-, di-, and trivalent ions showed that a choice in the range of 0.5–1.0 ps is more appropriate, with 0.5 ps being the recommended value.^37^ Following, the latter approach the MRT for a particular solvation shell is then given as
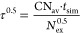
3with CN_av_ being the average CN of the respective solvation shell (obtained as the sum of the instantaneous CNs determined for each configuration of the simulation trajectory divided by the total number of configurations) and *t*_sim_ is the total simulation time. *N*_ex_^0.5^ corresponds to the total number of ligand exchange events registered along the simulation trajectory. The superscript 0.5 indicates the applied t*-value in ps. In addition to the MRT, the so-called rate coefficient *R*_ex_ proved to be a very useful property when comparing the solvation of different compounds^37^ and is defined as
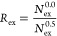
4with *N*_ex_^0.0^ being the total number of shell migration events in the absence of a minimum excursion time (i.e. t* = 0.0). Thus, *R*_ex_ can be interpreted as the average number of shell migration events required to achieve one sustained ligand exchange event lasting for *t* ≥ 0.5 ps.

The choice of the reference value of t* = 0.5 ps^37^ was inter alia influenced by the experimental value for the average H-bond lifetime in the pure solvent reported (at that time of publishing) as 0.55 ps.^22^ While this specific property has been intensely discussed over the last decades, similar values are also reported by different research groups for instance a value of 0.78 ps reported by Liu et al. in 2017.^53^ It should also be pointed out that MRT values of solvated ions span several orders of magnitude ranging from a few picoseconds, e.g., aqueous Cs^+^,^21^ to several hundred years, e.g., aqueous Ir^3+^.^5,6^ Thus, MRT values occur on a logarithmic scale, and the impact of considering a t*-value 0.5 or 1.0 ps is in practice very minor.

In order to further investigate the characteristics of short-time fluctuations in the CN over the simulations, the associated CN autocorrelation functions *C*_CN_(*t*) have been determined as
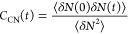
5with δ*N*(*t*) = *N*(*t*) – ⟨*N*⟩ being the CN fluctuation at a given time *t* and ⟨*N*⟩ represents the average CN.^54^ The associated relaxation time of the correlation function τ_CN_^54^ can then be obtained via the respective integral
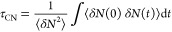
6

### 2.2 QM/MM MD Simulations

The ionic solutes considered in this work are Li^+^, Na^+^, K^+^, F^–^, Cl^–^, and Br^–^. The respective converged simulation trajectories were taken from earlier studies^41–43^ carried out using the using the in-house-developed QM/MM MD framework.^55,56^ In each case, a periodic, cubic simulation cell subject to the minimum image convention was employed, containing the solute and 2000 water molecules. The simulations were executed in the isobaric–isothermal (*NPT*) ensemble employing the Berendsen thermostat and manostat^57^ algorithms to maintain standard conditions (i.e., 298 K and 1.013 bar). The velocity-Verlet algorithm with a time step of 0.5 fs was employed to integrate the equation of motions.^58^ The size of the QM region was set in the range of 3.1–3.8 Å^41,42^ in case of the cations, while large QM radii ranging from 3.2–4.1 Å^43^ were employed in case of the anionic solutes. All simulation data were taken from previous studies^41–43^ with total simulation times of 100, 400, and ∼300 ps in the case of Li^+^, Na^+^, and K^+^ as well as ∼25 ps in the case of F^–^, Cl^–^, and Br^–^. The applied level of theory to treat interactions inside the QM region was resolution-of-identity second-order Møller–Plesset perturbation theory (RIMP2)^59–63^ as implemented in the TURBOMOLE package.^64^

In addition to simple ionic compounds, the solvation of a single carbon dioxide molecule in DCM as well as aqueous solution^50,51^ was considered, employing 1000 solvent molecules in each case. Again, the QMCF MD simulation trajectories obtained at the RIMP2 level taken from the previous studies were analyzed, with the total simulation times being 300 and 50 ps for CO_2_ in aqueous and DCM solution, respectively. The simulation protocol is similar to the one applied in the case of the ionic compounds discussed above with the size of the QM region being set to radii of 4.0 and 6.5 Å in the case of the aqueous and DCM solutions, respectively. For further details on the individual simulations analyzed in this study, the reader is referred to the original research articles.

### 2.3 DFTB MD Simulation of Pure Water

As a final test system, an MD simulation of pure water carried out at self-consistent charge density functional tight binding level of third order (DFTB3)^65^ was considered. The extension of the original 3ob parameter set^66,67^ enabling an improved treatment of aqueous systems (3obwp) reported by Goyal et al.^68^ was applied in conjunction with the D3 dispersion correction using Becke-Johnson damping.^69,70^ In addition, all interactions involving hydrogen were subject to an additional damping as required by the 3ob parametrization, setting the respective damping factor ζ to 4.0 au. A cubic simulation cell subject to periodic boundary conditions containing 250 water molecules was employed. Due to the large cell parameter of approximately 19.6 Å, a treatment considering only the Γ-point of the periodic system^71^ was considered adequate. The MD time step in the velocity-Verlet integration was set to 0.5 fs. The Berendsen thermostat and manostat algorithms^57^ were employed to maintain the target conditions of 298.15 K and 1.013 bar; the associated relaxation times were set to 0.1 and 10.0 ps, respectively. Initial equilibration in the *NVT* ensemble was carried out for 12.5 ps, followed by another 25 ps of re-equilibration under *NPT* conditions. Sampling was performed for 1,000,000 MD steps, resulting in a total simulation time of 0.5 ns. The simulation was performed using the in-house-developed QM/MM MD^55,56^ simulation program interfaced to the DFTB+ package.^72^

## 3 Results and Discussion

In this section, the data obtained for the CN and the associated ligand exchange dynamics for each system using the different NN algorithms are discussed, starting with the monovalent cationic and anionic solutes. In addition to characterizing the CNs, MRTs, and *R*_ex_ values, the separation of the first solvation shell from the remaining solvent molecules is evaluated by means of segmented RDF plots. Finally, an analysis of the instantaneous fluctuations in the number of ligands is carried out via CN autocorrelation functions. The more complex solvation properties of carbon dioxide in aqueous and DCM solutions are discussed next. As a final example, the solvation dynamics of pure water obtained from a 0.5 ns MD simulation carried out at the DFTB3 level is presented.

### 3.1 CNs of Ionic Hydrates

In order to investigate the coordination of the individual ionic solutes, the simulation trajectories of previous studies were re-evaluated using the GC, RAD, RAD_open_, and MV algorithms. Following the individual assignment of ligand molecules, transitions from the first solvation layer can be detected. By applying the direct algorithm,^37^ the average first shell coordination number CN_avg_ and the registered number of exchanges per ps *N*^0.5^ are determined (see Table 1).

**Table 1:** Cut-Off Distance *r*^*m*^ in Å Defining the First Solvation Shell in the GC Method, Average First Shell CN and Average Number of First Shell Ligand Exchange Events *N*_ex_^0.5^ in ps^–1^ Registered with a Minimum Excursion Time of t* = 0.5 ps Per Simulation Time Obtained Using the GC, RAD, RAD_open_, and MV NN Algorithms, Respectively

Ion	*r*^*m*^	CN_GC_	CN_RAD_	CN_RAD_open__	CN_MV_	*N*_GC_^0.5^	*N*_RAD_^0.5^	*N*_RAD_open__^0.5^	*N*_MV_^0.5^
Li^+^	2.75	4.1	4.1	4.1	5.2	0.18	0.18	0.18	0.47
Na^+^	3.30	5.5	5.5	5.5	6.9	2.18	2.15	2.15	2.47
K^+^	3.70	7.0	6.8	7.1	9.1	6.48	6.13	6.30	7.29
F^–^	3.36	4.8	5.2	6.1	9.0	4.50	4.73	5.75	8.65
Cl^–^	4.16	8.1	7.5	8.3	11.0	5.10	5.04	5.47	6.70
Br^–^	4.30	9.1	7.4	8.5	11.8	10.17	7.72	9.26	11.77

First, the results for the cationic solutes Li^+^, Na^+^, and K^+^ are discussed. Due to the ion-dipole orientation observed in the cation–water interaction, radial cutoff criteria are usually sufficient to define the solvation shells. This is particularly true for Li^+^ and Na^+^, in which case virtually identical CN and *N*^0.5^ values are observed using the GC, RAD, and RAD_open_ algorithms. In contrast, higher CNs and more frequent ligand exchanges are observed using the MV method in both cases.

The K^+^ ion represents a special case in this discussion as it has been associated with an enhanced ligand exchange dynamics due to its structure-breaking properties.^73–75^ Although this concept has been challenged in the past,^76,77^ it is typically associated with a perturbed hydrogen bond network in aqueous solution in combination with accelerated ligand exchange dynamics.^37^ When comparing the different CN and *N*^0.5^ values obtained for K^+^, small deviations can be observed between the GC, RAD, and RAD_open_ methods, while the MV approach again yields significantly higher values.

An entirely different picture is obtained for the anionic solutes F^–^, Cl^–^, and Br^–^ in aqueous solution, which is a direct consequence of the different ion–solute interactions realized via hydrogen bonds. In all cases, larger deviations in the average CNs and the *N*^0.5^ values are observed. While again the MV approach consistently gives larger values, no trend can be observed when comparing the GC results with those obtained from the two RAD approaches. In particular, for F^–^, the GC method gives a lower average CN, while in the case of Cl^–^ and Br^–^, higher CN_avg_ values are obtained. Previously, it has been shown that the CNs obtained via the RAD method are in better agreement to those obtained from experimental studies.^43^ This finding also rules out the RAD_open_ algorithm as it consistently gives higher CNs compared to those of the RAD and GC frameworks. The difference in the analysis methods can also be observed in the corresponding *N*^0.5^ values, indicating that the determinations of the CNs and the registered ligand exchanges are directly related. Similar to K^+^, also the bromide ion has been classified as structure-breaking by some authors,^74,78^ which can well explain the minor inconsistencies in the results observed between the different NN algorithms, foremost those observed in the number of registered ligand exchanges *N*^0.5^.

In order to determine which of the employed algorithms provides the best classification of first shell ligands, the respective CNs have been compared with reference data in the literature obtained from both experimental and theoretical investigations. The average CN of 4.1 obtained for Li^+^ using the GC, RAD, or RAD_open_ approach is in good agreement with theoretical estimations given in the range of 4.0–4.1 of^42,73,79–81^ and experimental results ranging from 4.0 to 5.0.^40,82,83^ In contrast, the value of 5.2 resulting from the MV analysis has to be considered as too high.

A similar picture is found in the case of Na^+^ and K^+^. Application of the GC, RAD, or RAD_open_ algorithms yields average CNs of 5.5 and 6.8–7.1, respectively. These values are in good agreement with the theoretical estimations^41,75,84^ and experimental measurements.^40,85–88^ Although the weaker ion–solvent interactions of K^+^ in aqueous solution result in some minor deviations in the average CN, the overall CN data is consistent, with the exception being again the MV approach, yielding notably higher CNs for both Na^+^ and K^+^, respectively.

As also discussed in a previous study,^43^ the differences in the first shell definitions have a notable influence in case of the anionic systems. The associated CN_RAD_ and CN_RADopen_ values of 5.2 and 6.1 for F^–^ agree best with the literature.^73,88,89^ The average CNs have been reported as 5.8, 5.1, and 4.6 based on classical, CP MD, and QM/MM MD simulations,^73,89^ while a higher value of 6.9 was found based on neutron diffraction with isotope substitution measurements.^88^ While the GC value of 4.8 is in a lower range of the reference data, the MV approach overestimates the average CN, yielding a value of 9.

In the case of Cl^–^, a different picture is obtained, with the RAD method resulting in the lowest CN of 7.5. All other approaches yield higher CN values in the range of 8.1–11, respectively. Experimental and theoretical reference data for the average CN of aqueous Cl^–^ are reported in the range of 7–7.1.^5,88,90^ and 5–6.8,^73,91–93^ respectively. Similarly, for Br^–^, the smallest CN of 7.4 is obtained from the RAD approach, while values ranging from 8.5 to 11.8 are found using the other methods. Again, the CN of 7.4 obtained for Br^–^ using the RAD method agrees best with estimates in the literature, which report CNs in the range of 6.5–7.6^6,94^ and 7.2^88,94–98^ based on theoretical and experimental estimates.

**Figure 2:**
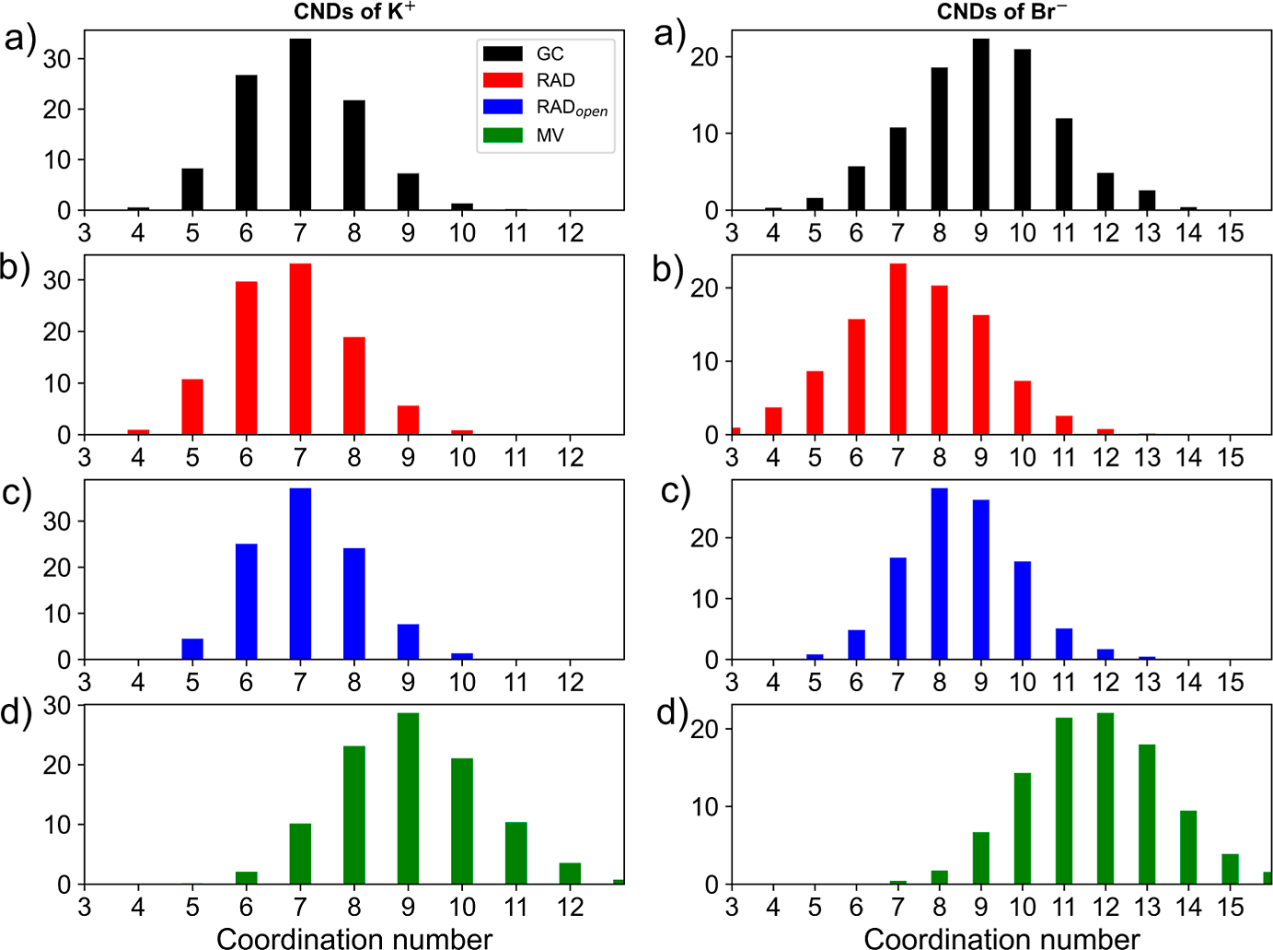
CNDs determined for aqueous K^+^ (left) and Br^–^ (right) obtained using the (a) GC, (b) RAD, (c) RAD_open_, and (d) MV NN algorithms, respectively.

To visualize the difference between the investigated NN algorithms, the CN distributions (CNDs) of the two most labile systems K^+^ and Br^–^ are shown in Figure 2. In both cases, a systematic increase in the CND plots can be observed, while the overall width of the distributions remains approximately constant with maximum probabilities ranging between 28 and 34% as well as 22 and 28% in the case of K^+^ and Br^–^, respectively. From these data, it appears that there is a systematic shift in the determination of the CN based on the applied NN used.

In order to assess whether the observed differences is indeed just due to a shift toward higher CN values, the Pearson and Spearman’s rank correlation coefficient for each pair of methods has been evaluated (see Supporting Information Table S1). From these data, several conclusions about the relationship between the different NN classification algorithms can be deduced. First, the systematic shift of the CNs along the sequence GC–RAD–RAD_open_–MV is well reflected by the individual Pearson and Spearman’s rank correlation coefficients observed for all investigated systems. Moreover, since the RAD and RAD_open_ methods are subtypes of the same general algorithm, one might expect that the best agreement would be found for these two approaches. However, the best overall agreement is found when correlating the GC approach with the RAD algorithm, both in terms of the Pearson and the Spearman’s rank correlation coefficient. Especially in the case of the cationic hydrates Li^+^, Na^+^, and K^+^, where the simple GC algorithm typically provides adequate data, the Pearson correlation and Spearman’s rank are found in the range of 0.57–0.86 and 0.58–0.85, respectively.

Together with the good agreement in the CNs, these data confirm that the RAD approach can provide an adequate classification of first shell ligands without the need for any predefined threshold. In contrast, the GC approach has been shown to give a less accurate NN assignment in case of the anionic compounds in the past.^43^ Nevertheless, the best overall correlation is found between the GC and the RAD approaches. Considering the comparatively simple implementation, it can be concluded that the RAD algorithm provides a general approach to classify direct solute–solvent contacts in the case of monatomic solutes. On the other hand, as already concluded from the overestimated average CN data resulting from the MV algorithm, this framework provides the least effective NN detection of the four tested algorithms.

### 3.2 Analysis of Shell Separation

**Figure 3:**
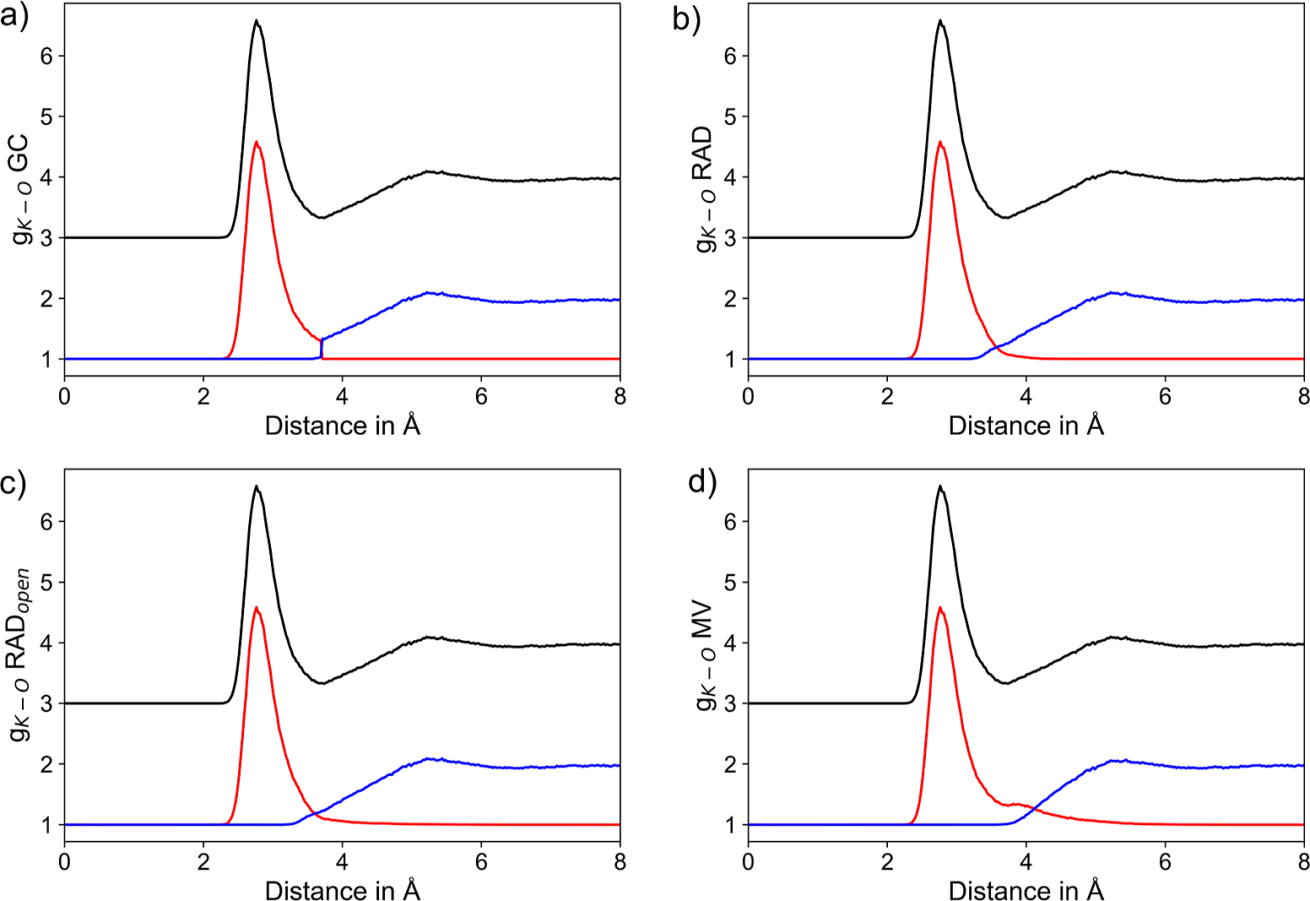
RDFs of K^+^–O_H_2_O_ pairs considering the entire RDF (black) along with the respective segmentation into the first solvation shell (red) and the remaining system (blue) based on the (a) GC, (b) RAD, (c) RAD_open_, and (d) MV methods, respectively. To improve the visibility, the total RDF is displayed at an offset of +3.

**Figure 4:**
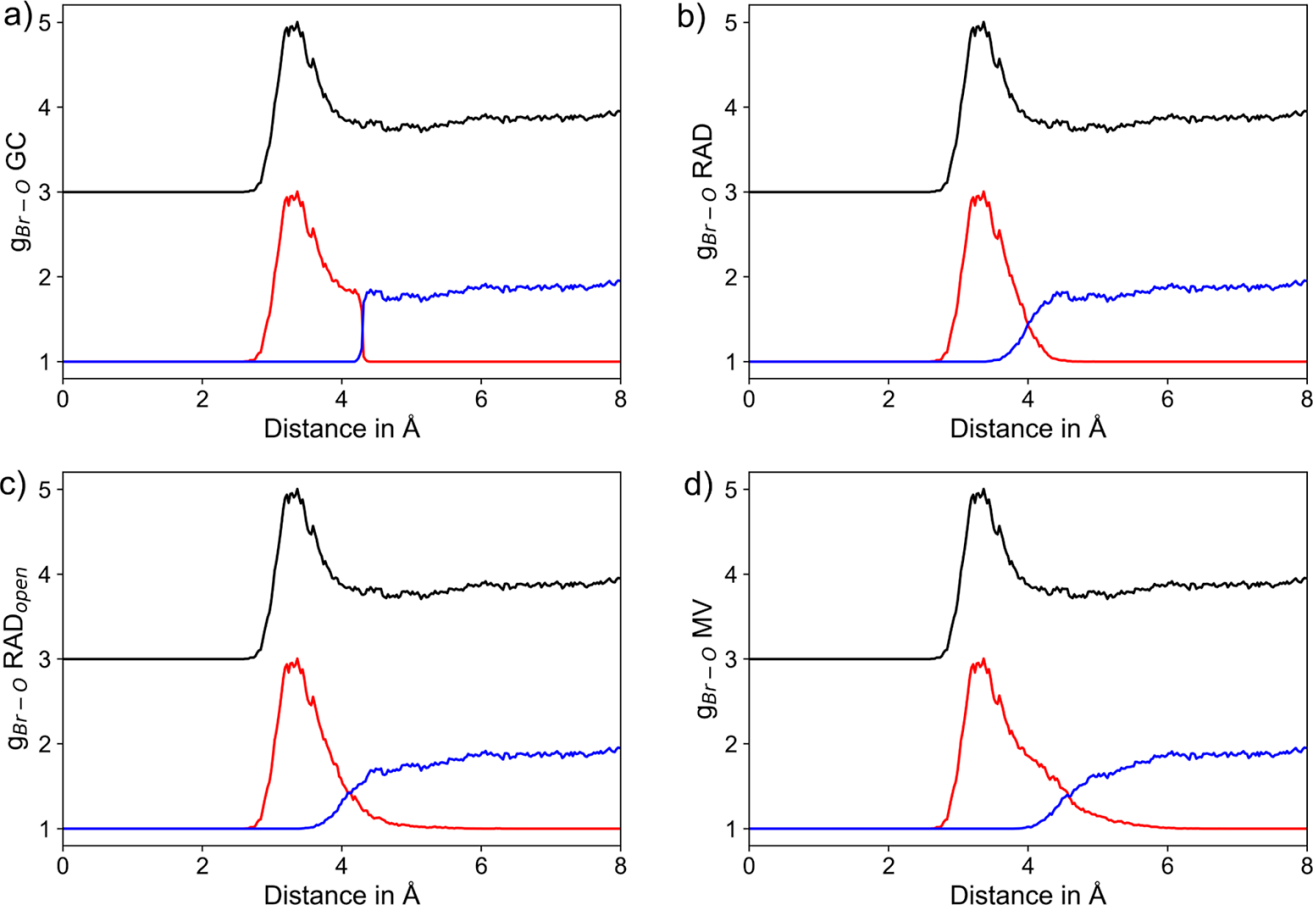
RDFs of Br^–^–O_H_2_O_ pairs considering the entire RDF (black) along with the respective segmentation into the first solvation shell (red) and the remaining system (blue) based on the (a) GC, (b) RAD, (c) RAD_open_, and (d) MV methods, respectively. To improve the visibility, the total RDF is displayed at an offset of +3.

In order to elucidate the separation between the first solvation shell and the remaining bulk, ion-O RDFs were prepared, which are exemplarily shown for the two most labile ionic solutes K^+^ and Br^–^ in Figures 3 and 4, taking into account the individual ligand assignment based on the four investigated algorithms. In case of the GC approach, a cutoff distance must be specified, which typically coincides with the minimum distance *r*^*m*^ separating the first shell from the remaining solvent. The corresponding values are listed in Table 1.

The comparison of the segmented K^+^–O RDFs obtained from four different NN algorithms is shown in Figure 3. In all cases, the dominant first shell peak located at a maximum distance of 2.8 Å is perfectly resolved. In contrast to the GC method, where the separation of the first shell RDF follows a step function (see Figure 3a), an overlap of the individual RDFs in the transition region can be observed (see Figure 3b–d) in case of the other NN algorithms. This implies that in some cases, ligands within the GC cutoff are excluded from the first shell, while on the other hand, solvent molecules located outside this cutoff may be considered as part of the first solvation layer.

The distance of the respective intersections between the two sub-RDF plots can be interpreted as an estimate of the shell boundary. Similar values of 3.57 and 3.61 Å are found using the two RAD approaches, while the point of intersection is found at 4.12 Å with the MV method. In the latter case, a small side peak in the range of 3.6–4.0 Å is observed. This density contribution to the RDF clearly results from second shell ligands, which are erroneously considered as NNs. The latter behavior is also visible in case of the more strongly hydrated Li^+^ and Na^+^ systems (see Supporting Information Figures S1 and S2), where the GC- and RAD-based methods achieve an ideal separation of the first solvation shell.

Despite the different solute–solvent interactions realized via hydrogen bonding, similar conclusions can be drawn when comparing the different approaches applied to the anionic hydrates. Figure 4 shows the segmented RDFs obtained for the hydrated Br^–^ system, which is the most weakly hydrated ionic system considered in this study. As before, the separation in the GC method corresponds to a step function, and again both RAD approaches result in a well-separated first solvation shell peak near 3.3 Å. The intersection of the segmented RDF plots resulting from the RAD, RAD_open_, and MV algorithms can be found as 4.0, 4.1, and 4.58 Å, respectively. In the latter case, the notable shoulder in the segmented RDF of the first shell again indicates the inclusion of second shell ligands in the NN assignment. Identical conclusions can be drawn when considering the segmented RDFs obtained for F^–^ and Cl^–^ shown in the Supporting Information Figures S3 and S4, respectively.

In addition to the discussion of the individual shell boundaries resulting from the different NN methods, the area resulting from the overlap of the two segmented RDF contributions resulting from the application of the RAD, RAD_open_, and MV approaches has been determined via numerical integration using the trapezoidal rule (see Supporting Information Figure S5 and Table S2). In all cases, the RAD method gives the smallest area, indicating that this approach results in the least overlap in the separation between the first shell and the remaining solvent, making the RAD approach the preferred choice in this regard.

### 3.3 Dynamics of Ligand Exchange

The differences in the average CNs discussed above are also reflected in the average number of exchanges per ps *N*^0.5^ also listed in Table 1. While consistent estimates are obtained for the cations, the anionic solutes show notable deviations. In both cases, the most weakly hydrated ions K^+^ and Br^–^ also show the largest deviations between the different algorithms. Again, the MV approach consistently results in higher values, which, together with the too high CNs, result from an inadequate assignment of ligands. As seen from the segmented RDFs, ligands of the second solvation layers are incorrectly assigned to the first shell, and it can be assumed that the number of exchanges *N*^0.5^ wrongly takes intrashell mobility of second shell ligands into account.

Despite the remarkable differences observed in the first shell ligand assignment leading to significant variations in the average CNs, the segmented RDFs and the number of registered ligand exchanges, the evaluation of the MRT τ^0.5^ yields surprisingly consistent results (see Table 2). The only notable outlier for the investigated systems is the value of 11 ps obtained for Li^+^ in conjunction with the MV algorithm, while all other approaches yield values of 23 ps. However, for both Na^+^ and K^+^ as well as for all anionic solutes, the MRT values obtained are in very good agreement when comparing the different algorithms used. Even the estimates obtained using the MV approach show only very minor deviations compared to the GC and RAD methods.

**Table 2:** MRT τ^0.5^ in ps and the Associated Rate Coefficient *R*_ex_ Determined via the Direct Method Using the GC, RAD, RAD_open_, and MV NN Algorithm, Respectively

ion	τ_GC_^0.5^	τ_RAD_^0.5^	τ_RAD_open__^0.5^	τ_MV_^0.5^	*R*_ex_^GC^	*R*_ex_^RAD^	*R*_ex_^RAD_open_^	*R*_ex_^MV^
Li^+^	23	23	23	11	3.77	18.95	22.83	65.64
Na^+^	2.5	2.5	2.6	2.8	1.81	2.97	3.65	9.47
K^+^	1.1	1.1	1.1	1.2	1.57	2.78	2.99	4.07
F^–^	1.1	1.1	1.1	1.1	5.21	7.67	6.50	5.62
Cl^–^	1.6	1.5	1.5	1.6	4.48	6.69	4.49	5.53
Br^–^	0.9	0.9	0.9	1.0	2.93	6.93	4.43	3.83

This unexpected result needs to be discussed in the context of the determination of the MRT. Whenever a ligand is assigned to a different solvation layer compared to the previous configuration in the trajectory (either by crossing the boundary of the first solvation layer *r*^*m*^ or by a more advanced NN determination such as the RAD or MV approaches), its further trajectory is monitored. If the displacement from its original shell lasts longer than the minimum excursion time, t*, the event is considered as a sustained exchange process. The ligand is then assigned to its current solvation shell and monitored again if a further change in the solvation layer assignment is registered. By monitoring the intershell migration of all ligands over the entire simulation trajectory, the associated MRT value can be determined as the product of the average CN in the solvation shell times the total simulation time divided by the total number of registered ligand exchanges with a displacement time of t* ≥ 0.5 ps (see eq 3). Obtaining consistent estimates for the MRT values, despite the considerable differences in the average CNs, implies that these variations are compensated to a large extent by the number of registered exchanges. It can therefore be concluded that the determination of the MRTs is largely independent of the method used to assign ligands to the different solvation shells.

This finding holds great promise for the analysis of solvation dynamics from MD simulations as it greatly simplifies the computational workflow. Despite the inadequate CN resulting from the much simpler radial cutoff in case of anionic solutes, the analysis of the associated exchange dynamics can still be carried out using a simple GC-based shell assignment. This greatly simplifies the associated implementation of the direct algorithm^37^ for the determination the MRT, while the resulting error appears to remain within an acceptable margin (e.g., 1.6 vs 1.5 ps when comparing the GC and RAD approaches in case of the highly labile aqueous Br^–^ system, see Table 2).

In addition to the mean residence time, the rate coefficient *R*_ex_ was introduced as a further key property to characterize the dynamics of ligand exchange from simulation data.^37^ It is defined as the ratio of the total number of registered intershell transitions *N*^0.0^ regardless of the actual excursion time (i.e., t* = 0.0) and the number of ligand exchanges *N*^0.5^ persisting for the minimum excursion time t* = 0.5 ps (see eq 4). Thus, *R*_ex_ can be interpreted as the average number of intershell crossings required to achieve an actual sustained ligand exchange from/to the solvation shell. The rate coefficients obtained for the six ionic solutes using the four different algorithms are listed in Table 2. In contrast to the CNs and the MRT data, the individual estimates show strong deviations between the individual NN classifications. Based on the highly similar MRT values obtained for the different methods, it can be concluded that the differences in *R*_ex_ can be explained by strong variations in the *N*^0.0^ values that are listed in the Supporting Information Table S3. It can be seen that the GC approach shows the smallest values in *N*^0.0^. This implies that considering first shell ligands on the basis of a radial cutoff leads to the smallest number of short time intershell crossings per exchange event, while for all other methods, notably larger values are found. This finding is not too surprising when considering the overlapping contributions in the segmented RDF plots discussed above. It can be expected that a larger number of short time shell transitions occurs in the overlap region. However, as seen from the MRT values, these short-time fluctuations in the shell assignment have only a very little effect on the evaluation of the ligand exchange dynamics since the choice of a sensible minimum excursion time t* eliminates the impact of these short-lived perturbations to the solvation structure. It should also be noted that due to the large deviations in *R*_ex_ found between the different NN algorithms, the interpretability of *R*_ex_ can be debated, and it remains to be seen to what extent this property will be useful in future studies of ligand exchange dynamics.

CN autocorrelation functions *C*_CN_(*t*) and the associated relaxation time τ_CN_ are another commonly employed method to characterize variations in the coordination of a solvated species.^54^ In general, autocorrelation functions measure the extent to which a signal correlates with its own copy at a later stage. Low values for the relaxation time indicate a fast loss of information due to a low correlation in the signals, i.e., the variation in coordination at a later time is effectively unrelated to that in the early phase of the simulation. In this context, it is of particular interest whether the differences in the number of total fluctuations *N*^0.0^ obtained using the different NN algorithms are associated with different relaxation times in the *C*_CN_(*t*).

**Figure 5:**
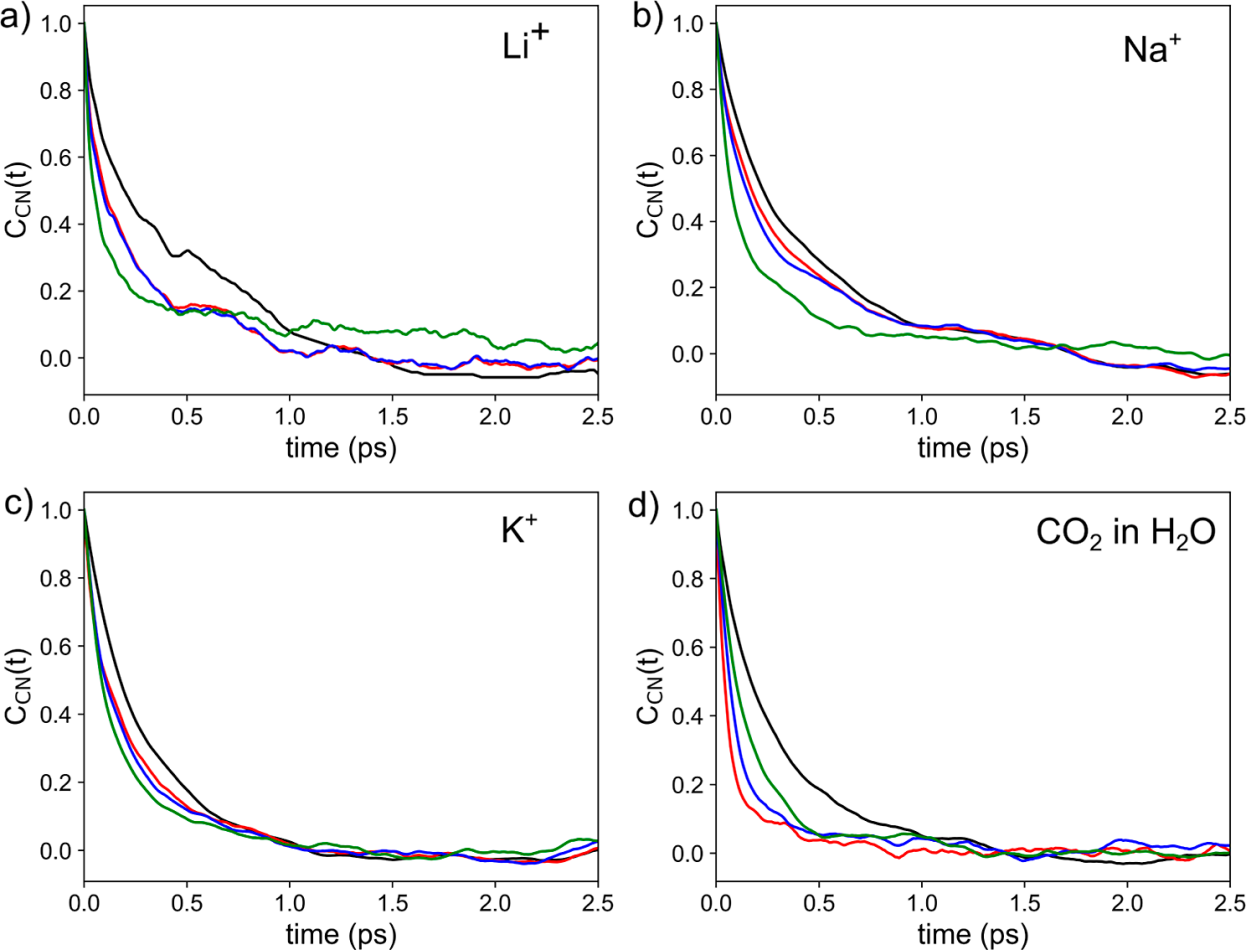
CN autocorrelation function *C*_CN_(*t*) determined for (a) Li^+^, (b) Na^+^, (c) K^+^, and (d) CO_2_ in aqueous solution obtained using the GC (black), RAD (red), RAD_open_ (blue), and MV (green) NN algorithms.

The autocorrelation functions *C*_CN_(*t*) determined for Li^+^, Na^+^, and K^+^ in aqueous solution obtained using GC, RAD, RAD_open_, and MV are depicted in Figure 5a–c; the respective associated relaxation times τ_CN_ are listed in Tab. S6. Overall, a steep decay of *C*_CN_(*t*) within the first ∼0.5 ps can be observed, which effectively approaches zero after approximately 1.0–2.0 ps. The largest estimates for the τ_CN_ are observed in the case of the GC algorithm, with the respective values being 0.32, 0.35, and 0.24 ps for Li^+^, Na^+^, and K^+^, while the other NN algorithms yield smaller values in the range of 0.21–0.29, 0.22–0.19, and 0.18–0.19, respectively. These data align well with the different values in *N*^0.0^ (Table S4), although the difference are much less pronounced. Based on these values, it can be concluded that the applied value for the minimum excursion time t* of 0.5 ps is indeed a suitable choice as it strikes a highly suitable balance in eliminating the influence of short-time fluctuations in the coordination while being sufficiently short to discriminate the MRTs of different species. These two aspects were the main consideration when introducing this value in the original publication.^37^

This conclusion is further underlined by the respective data obtained for F^–^, Cl^–^, and Br^–^ in aqueous solution (see Figure S6a–c and Table S6). Although a higher degree of noise in the *C*_CN_(*t*) plots is observed, the general trend of a steep decline within the first 0.5 ps is still visible. In all cases, much smaller relaxation times than in case of the cationic solutes are observed, except for the RAD_open_ and MV approaches applied to the Br^–^ system. However, these two algorithms have already been ruled out in terms of the structural analysis discussed above. On the other hand, the GC and RAD algorithms show highly similar estimates for τ_CN_ being in the range of 0.07–0.08 ps for F^–^ and Cl^–^ as well as 0.11–0.14 in case of Br^–^. These values reflect that due to faster ligand exchange dynamics seen in the MRT values, correlations in the time evolution of the CN decay even faster than in the case of the more stable cationic hydrates. Thus, also in this case, the recommended t* of 0.5 ps appears adequate to separate persistent ligand transition events from/to a particular solvation shell and the associated short-time fluctuations.

Finally, the exceptionally low MRT values of the various systems on the picosecond scale should be addressed. In previous studies,^20,21,73,75^ high exchange rates, especially those being faster than the MRT of pure water reported in the range of 1.51–2.45 ps,^36^ have been associated with structure-breaking properties. According to a detailed discussion provided by Marcus,^74^ only Li^+^ is classified as a structure-forming species, K^+^, Cl^–^, and Br^–^ are considered as structure-breaking, and Na^+^ and F^–^ are borderline in between these two extremes. The concept of structure-making and -breaking has been challenged by several authors^76,77^ based on the fact that these effects do not have a consistent impact on specific observables such as the vibrational and reorientational dynamics of water, hydrogen bond lifetimes, or structural properties. In this context, the picosecond water exchange dynamics in a given solvation shell (which still is highly challenging to determine experimentally) provides a promising rationale to explain this effect based on an acceleration or deceleration of the ligand exchange dynamics with respect to the pure solvent. Nevertheless, a recent study by Reynolds has shown that the concept of structure-making and -breaking can be directly related to the associated Lewis acidity/basicity of the solvated species relative to pure water,^77^ which is a promising candidate to replace the previously adopted classification scheme with a more modern approach.

### 3.4 Solvation Dynamics of CO_2_

The systems considered in the examples discussed above only consider a monatomic ion in aqueous solution. In order to assess the performance of the different NN algorithms when applied to more complex, polyatomic solutes, the simulation trajectories of two previous QM/MM MD studies of carbon dioxide in aqueous^50^ and DCM^51^ solution were analyzed.

Carbon dioxide is a highly suitable test case since it combines cationic and anionic interaction motifs in a single moiety. More specifically, the interaction with the solvent can either be realized via hydrogen- or halogen-bonding at the oxygen atoms of CO_2_, which is analogous to the solvation of anions. In addition, due to the positive partial atomic charge of the carbon atom, a solute–solvent charge–dipole interaction is observed, similar to the case of cationic solvation. In previous studies,^50,51^ the characterization of the solvation structure required a series of several individual analysis steps, including the calculations C- and O-solute pair distribution functions, an intricate projection procedure to monitor the solvent density distribution with respect to the molecular axis^31^ followed by the calculation of segmented pair distributions. This enabled the assignment of solute molecules in the vicinity of the carbon and oxygen atoms, respectively, and it is of particular interest whether a similar assignment of ligands to the first solvation shell can be achieved when using the RAD, RAD_open_, and MV algorithms.

**Figure 6:**
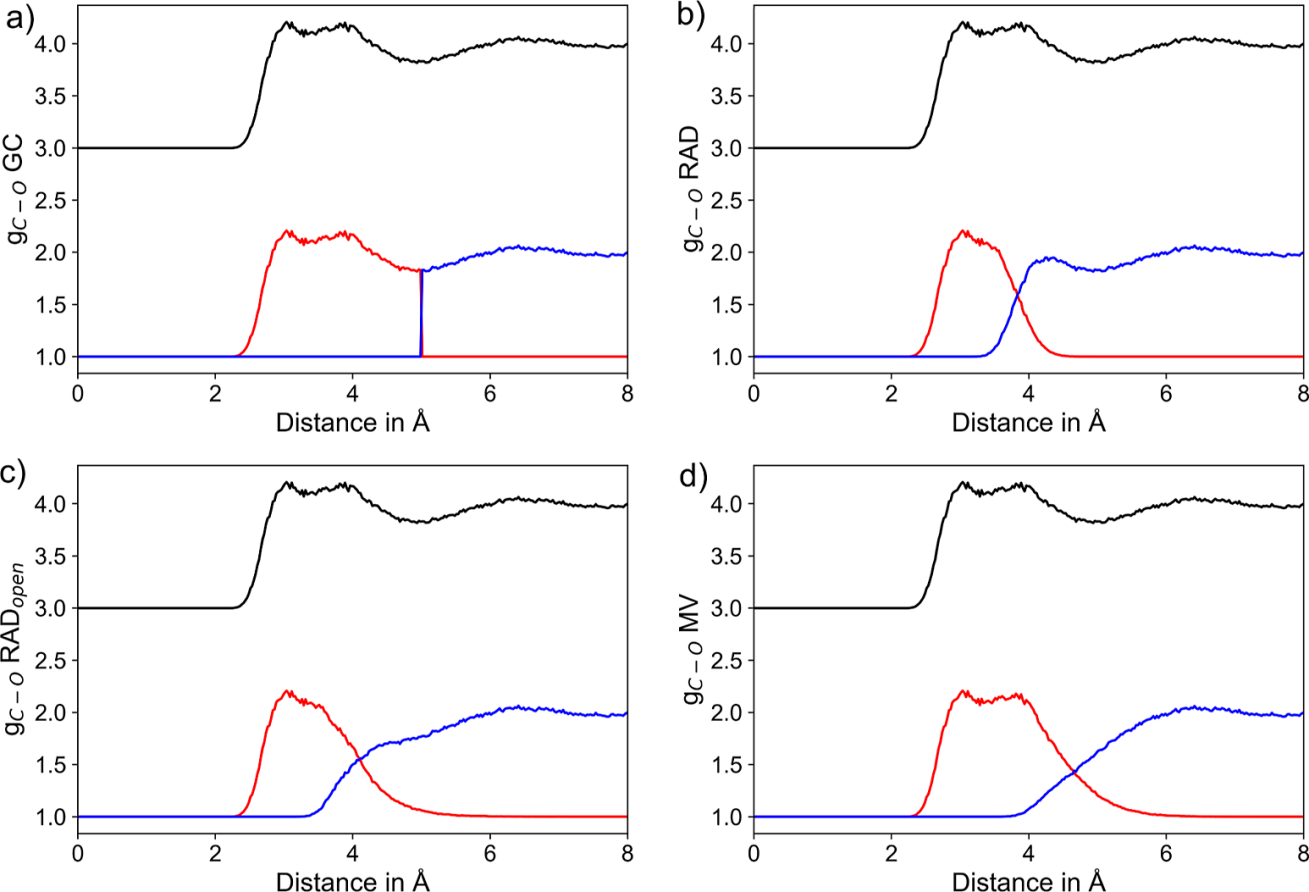
RDFs of the C_CO_2__–O_H_2_O_ pairs considering the entire RDF (black) along with the respective segmentation into the first solvation shell (red) and the remaining system (blue) based on the (a) GC, (b) RAD, (c) RAD_open_, and (d) MV methods, respectively. To improve the visibility, the total RDF is displayed at an offset of +3.

The respective CNs of CO_2_ in water evaluated by the GC, RAD, RAD_open_, and MV algorithms are listed in Table 3. It can be seen that the values obtained via the two RAD-based approaches of 6.0 and 8.3 are significantly smaller compared to those of the GC and MV methods found as 14.5 and 12.3, respectively. This discrepancy can be explained by comparing the segmented C–O_H_2_O_ RDFs shown in Figure 6. In the case of the two RAD-based methods, the distance of intersection of the segmented RDFs is found at 3.83 and 4.10 Å, respectively, whereas a larger value of 4.65 Å is obtained when using the MV framework. The segmentation resulting from the RAD approach mainly considers water molecules close to the carbon atoms, i.e., a separation of the solvation based on an atom-based criterion (see Figure 6d). Indeed the associated segmented RDFs agree well with those of conically segmented RDF previously reported,^50^ which were obtained by preselecting solvent molecules based on an angular criterion with respect to the molecular axis.

**Table 3:** Cut-Off Distance *r*^*m*^ in Å Defining the First Solvation Shell in the CO_2_-Solvent RDF, Average First Shell CN and First Shell MRT τ^0.5^ in ps Determined via the Direct Method Using the GC, RAD, RAD_open_, and MV NN Algorithms, Respectively

solute	solvent	*r*^*m*^	CN_GC_	CN_RAD_	CN_RAD_open__	CN_MV_	τ_GC_^0.5^	τ_RAD_^0.5^	τ_RAD_open__^0.5^	τ_MV_^0.5^
CO_2_	H_2_O	5.0	14.5	6.0	8.3	12.3	1.5	1.3	1.3	1.4
CO_2_	DCM	6.5	8.8	6.3	7.2	9.6	2.5	2.5	2.5	2.8

In contrast, the MV algorithm achieves an ideal separation of the entire solvation shell with the respective intersection distance being ideally aligned with the corresponding minimum in the overall C–O_H_2_O_ RDF (see Figure 6b). The NN classification resulting from the RAD_open_ method lies between those of the RAD and MV frameworks and appears to be the least suitable option in this case. The integration of the area of overlap between the segmented RDFs is shown in Figure 6. In contrast to the ionic solutes discussed above, the area resulting from the MV method is similar to those obtained from the RAD-based NN classification, indicating that the MV approach is more efficient in the case of a molecular solute.

To explain why the RAD algorithm underestimates the number of water molecules in the first solvation shell of CO_2_, an example configuration taken from the simulation is shown in the Supporting Information Figure S7. In this case, five water molecules surround the positively charged central carbon of the CO_2_ molecule and interact mainly via charge–dipole interactions. When considering the ligand in the proximal position of CO_2_ near the O atom at a distance of *r*_*ij*_, these five ligands are considered at a distance labeled as *r*_*ik*_ in the RAD approach (*cf*Figure 1). Depending on the angle θ_*jik*_, it is indeed possible that the RAD condition (eq 1) excludes the proximal ligand due to the large difference between *r*_*ij*_^2^ and *r*_*ik*_^2^ (see eq 1). This suggests that the RAD algorithm is less suitable in the case of composite solutes such as CO_2_ as ligands that are clearly part of the first contact layer may be excluded from the first shell ligand assignment.

As might be expected, the differences in the CNs obtained from the different approaches are well reflected in the registered number of exchanges, also listed in Table 3. However, even for this more complex solute, the four different algorithms give very similar MRT values in the range of 1.3–1.5 ps. In contrast, the values for *R*_ex_ again show large deviations as already observed in case of the monatomic solutes discussed above. The observed differences can be associated with the stronger solute–solvent interaction near the carbon site as an average of 12.3 short-time fluctuations occur before a sustained ligand exchange is achieved when using the RAD approach. In contrast, the GC and MV approaches show significantly smaller values of 4.1 and 5.2 (see Supporting Information Table S4), respectively. This suggests that on average only a few exchange attempts are required to achieve a prolonged ligand exchange, which is a result of the weaker solute–solvent interaction near the O atoms of carbon dioxide.^50^

**Figure 7:**
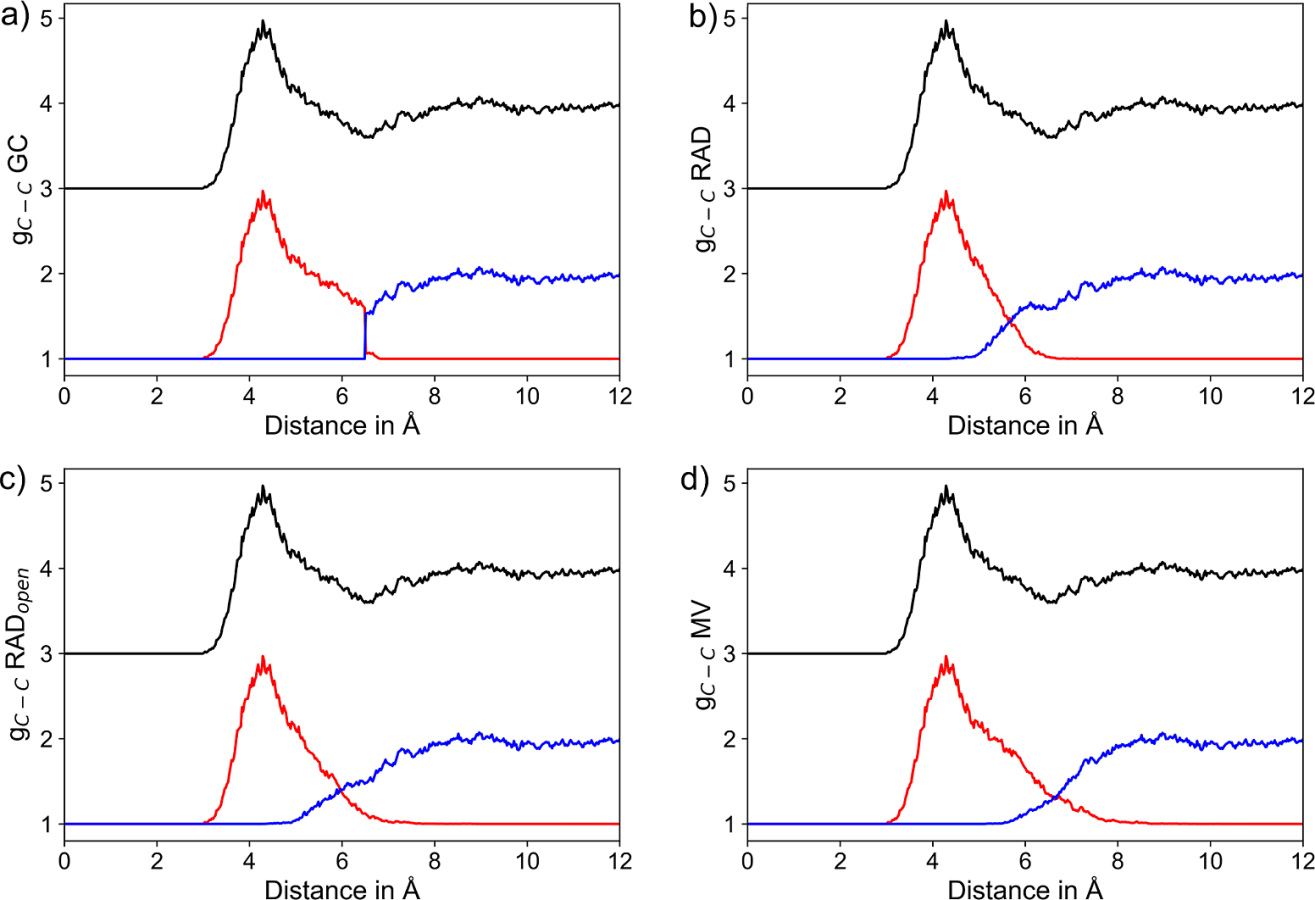
RDFs of the C_CO_2__–C_DCM_ pairs considering the entire RDF (black) along with the respective segmentation into the first solvation shell (red) and the remaining system (blue) based on the (a) GC, (b) RAD, (c) RAD_open_, and (d) MV methods, respectively. To improve the visibility, the total RDF is displayed at an offset of +3.

In the case of CO_2_ in DCM solution, a similar performance of the NN algorithms is observed. However, due to the larger volume of the DCM molecules, the differences are not as pronounced as in the aqueous case. Again, the RAD algorithm gives the lowest CN and *N*^0.5^ values, while the GC and MV approaches produce comparable data. Also in this case, the intersection of the segmented RDFs shown in Figure 7 resulting from the MV method aligns ideally with the minimum in the C_CO_2__–C_DCM_ RDF, while both RAD approaches show an intersection at smaller distances.

As already observed for different examples discussed above, the differences in the ligand assignment again have very little influence on the MRT (see Table 3). A value of 2.5 ps was obtained when using the GC, RAD and RAD_open_ algorithms, while a slightly higher estimate of 2.8 ps was determined using the MV framework. For the rate coefficient *R*_ex_, a trend similar to that observed for aqueous solution was observed, with higher values being found in the case of the RAD approaches. As before, a reduction of *R*_ex_ is observed when applying the GC and MV algorithms since the inclusion of the entire solvation layer, considering also the coordination near the O_CO_2__ atoms, leads to an overall weaker interaction on average.

The correlation function of the fluctuations in *C*_CN_(*t*) of CO_2_ in H_2_O obtained using the GC, RAD, RAD_open_, and MV algorithms are depicted in Figure 5d. As in case of the ionic hydrates discussed above, a fast decay of *C*_CN_(*t*) within the first 0.5 ps is observed in case of the RAD and GC approach in case of CO_2_ in H_2_O, while a slower decay is observed for CO_2_ in DCM (see Figure S6d). The GC algorithm again leads to the slowest decay in both cases, followed by the MV, RAD, and RAD_open_ approaches. Table S7 lists the relaxation time of the autocorrelation function τ_CN_ for CO_2_ in H_2_O and DCM obtained using the different algorithms. In the case of CO_2_ in H_2_O, it can be seen that the τ_CN_ obtained via the two RAD-based approaches of 0.10 and 0.16 ps are smaller compared to those of the GC and MV methods found at 0.27 and 0.18 ps. Since the structural analysis has shown that the RAD-based algorithms are not capable of fully describing the entire solvation of the molecular structure, these data have to be considered as less reliable. Although the τ_CN_ value obtained in the case of the MV approach is notably smaller compared to the GC method, all decay constants are below the threshold for t* set to 0.5 ps, implying that also in this case, the correlation in the short-time fluctuation decays faster than the preset value for the minimum excursion time.

In contrast to these findings, the case of CO_2_ in DCM is, to some extent, uncertain. The corresponding *C*_CN_(*t*) plots displayed in Figure S6d show a notably longer decay compared to the aqueous systems discussed above. In the original work,^37^ the value of t* was inter alia based on the experimental value for the average H-bond lifetime in pristine H_2_O. It is, therefore, unclear whether this value also provides a good reference for studying ligand exchange in DCM solution.

When considering the largest decay constants obtained for CO_2_ in DCM in the case of the GC and MV algorithms amounting to approximately 0.5 and 0.46 ps, it appears that the t* value of 0.5 ps is indeed in the borderline range. However, when also considering the much higher mass of a CH_2_Cl_2_ molecule being about 84.9 g mol^–1^, the motion of DCM molecules in solution can be expected to be much slower compared to H_2_O. This is directly reflected by the associated *N*^0.5^ and *N*^0.0^ values, being notably smaller in the DCM case, pointing to a much smaller degree of short-time exchange events (see Supporting Information Tables S4 and S5).

Thus, based on the available data, the t*-value of 0.5 ps appears to represent a suitable threshold for investigating ligand exchange dynamics in the DCM solution as well. In order to provide further insights into the native ligand dynamics, a long time simulation of DCM would have to be carried out. However, due to the more complex nature of this solvent (a single DCM molecule contributes a total of 42 electrons, 20 of those being in the valence shells), such a simulation is much more challenging when considering a quantum chemical or semiempirical level of theory.

In summary, it can be concluded that the MV approach provides a better separation of the NNs from a molecular perspective, while the RAD approach is suitable to identify NNs with respect to the central atom of the investigated solute. Since a water molecule can simultaneously act as a donor as well as an acceptor for hydrogen bonds, pure liquid water represents a particularly challenging test case, which is discussed in the following.

### 3.5 Solvation Dynamics of Water

Water plays an essential role in a large number of chemical processes, and its properties are inter alia closely related to the dynamics of its solvation shell, which is known to occur on the picosecond scale. The high electronegativity of the oxygen atom leads to a dominant intermolecular polarization of the water molecule, which is responsible for its overall highly polar nature. As a result of the strong dipole moment, water is therefore able to donate hydrogen bonds via the positively charged H atoms and to accept H-bonds at the negatively charged O atom. As many chemical reactions take place in aqueous environments, these features give water exceptional properties at the center of chemical and biological processes. A large number of studies have investigated the solvation dynamics of water,^5,6,36,99–104^ which proved to be a very challenging topic due to the very fast ligand exchange rate. It was, therefore, of particular interest to study the coordination properties and the respective exchange dynamics using different NN algorithms in order to gain a new perspective on the study of these critical properties of water.

A particularly promising approach to investigate aqueous systems at the molecular level is density functional tight binding of third order (DFTB3), in particular the extension of the original 3ob parameter set^66,67^ developed by Goyal and co-workers optimized for the description of water.^68^ DFTB methods can be considered as a simplified variant of density functional theory (DFT). Despite being heavily parametrized, DFTB still retains the key features of a quantum chemical many-body description of the associated electron density. By introducing a suitable parametrization, the semiempirical DFTB approach was shown to provide data of similar accuracy as DFT in many cases,^105–111^ while the associated execution times are 2–3 orders of magnitude faster. This enables MD simulations of comparably large systems for long simulation times. In the present case, a periodic unit cell containing 250 water molecules was simulated for a total sampling time of 0.5 ns at ambient conditions.

The intermolecular O–O, O–H, and H–H RDFs obtained from the DFTB MD simulation are shown in the Supporting Information Figure S8, indicating a very good agreement with experimental estimates of.^112–114^ Similarly, the density profile resulting from the *NPT*-based simulation is in good agreement with the experimental density being 0.997 kg dm^–3^ (see Supporting Information Figure S9). Since the dynamics of ligand exchange are represented in the O–O RDF via the absolute values of the maxima and minima,^115^ it can be concluded that the simulation shows a very similar exchange rate compared to the experimental measurements. By considering a different water molecule as the central species, the MRT and CN_av_ value can be evaluated several times based on the simulation data. Thus, in this case, a total of 250 individual estimates can be made, providing detailed insights into the variation of each property.

**Figure 8:**
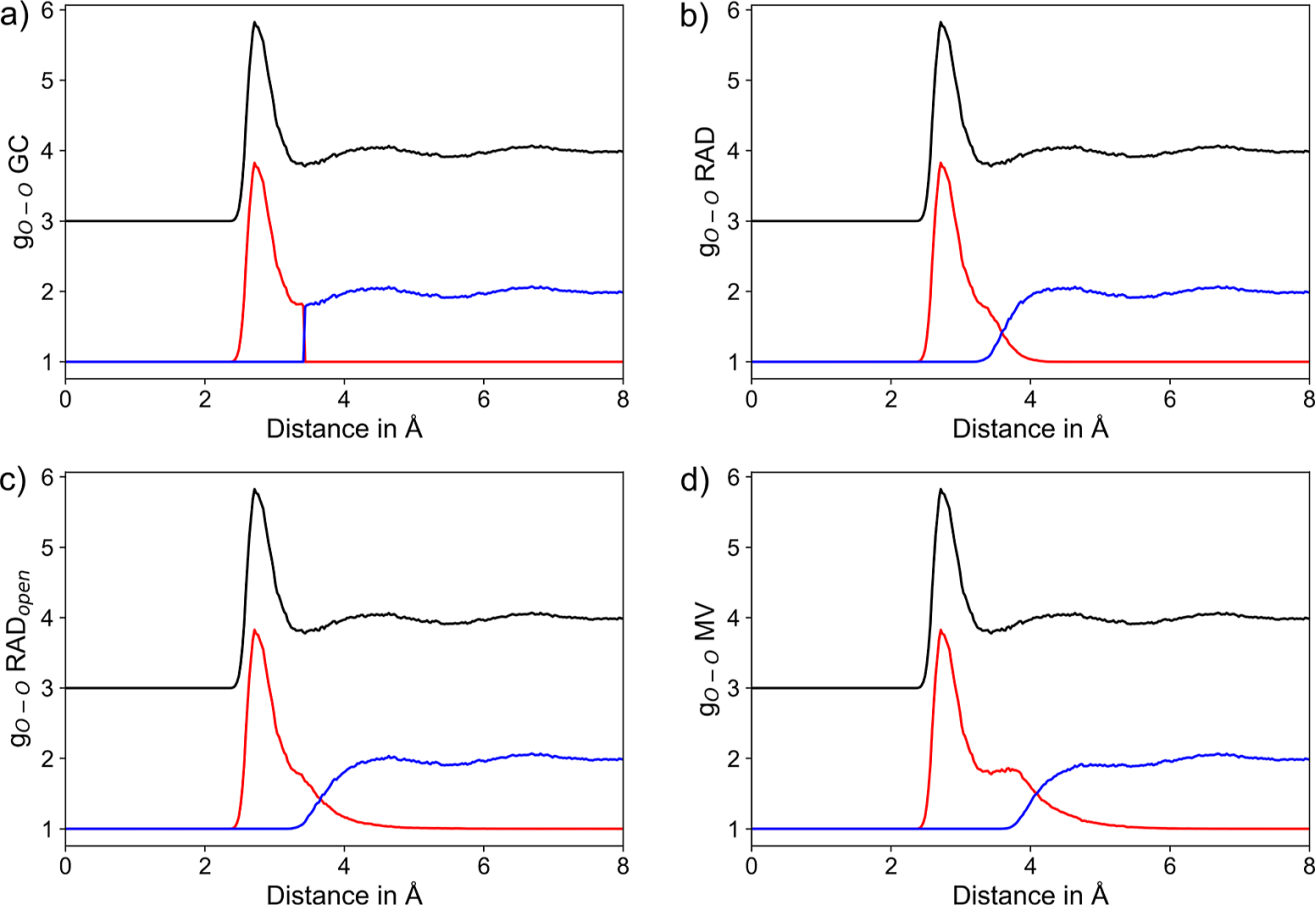
RDFs of the O_H_2_O_–O_H_2_O_ pairs considering the entire RDF (black) along with the respective segmentation into the first solvation shell (red) and the remaining system (blue) based on the (a) GC, (b) RAD, (c) RAD_open_, and (d) MV methods, respectively. To improve the visibility, the total RDF is displayed at an offset of +3.

A comparison of the average CN determined for each of the 250 water molecule using the GC, RAD, RAD_open_, and MV algorithms are shown in Supporting Information Figure S10. The respective mean values and standard deviations obtained by averaging over all water molecules are listed in Table 4. When comparing the results obtained for different H_2_O molecules obtained using a specific NN algorithm, consistent estimates are obtained and the respective standard deviations are comparably small (see Table 4). Similar to the ionic solutes discussed above, the individual algorithms yield largely differing CNs, with both the RAD-based methods as well as the MV approach clearly overestimating the CN expected for a water molecule in the pristine solvent. Evaluation of the segmented O–O RDFs obtained by choosing a random water molecule as center species (see Figure 8) shows that the MV algorithm tends to include a large portion of the second shell into the first shell definition similar as observed in case of the monatomic solutes discussed above. While this is largely reduced in the RAD-based methods, in both cases, a notable shoulder toward larger distances is observed in the first shell RDFs, which explains the increased CNs of 6.01 and 6.95. Thus, in this case, a radial cutoff as realized in the GC framework appears to be the most reliable approach, yielding a CN of 5.10 when considering the first shell minimum distance of 3.4 Å taken from the respective O–O RDF. This is still larger compared to the expected value reported in the range of 4.5–5^36,116–118^ and can be attributed to shortcomings in the semiempirical nature of the applied DFTB3 approach. However, as recently highlighted by Willow et al., the CN of water is on average higher than the actual number of registered hydrogen bonds.^117^ This finding can be explained by ligands that are assigned to the first solvation shell but are not H-bonded to the respective central water molecule as they are in the initial of the final stage of ligand exchange.

**Table 4:** Average First Shell CN and First Shell MRT τ^0.5^ in ps of Pure Water Determined via the Direct Method Using the GC, RAD, RAD_open_, and MV NN Algorithms, Respectively^a^

	GC	RAD	RAD_open_	MV
CN_av_	5.10 ± 0.038	6.01 ± 0.044	6.95 ± 0.047	10.18 ± 0.064
τ^0.5^	1.46 ± 0.036	1.56 ± 0.036	1.56 ± 0.034	1.60 ± 0.027

a The average values and the associated standard deviations are obtained by averaging over all 250 water molecules in the system.

The mean values and standard deviations of the MRT obtained from the four different NN algorithms via averaging over all 250 water molecules are listed in Table 4. Similarly, as shown in the examples above, the obtained MRT values are effectively independent with respect to the applied algorithm. The values found in the range of 1.46–1.60 ps (see Figure S11) agree well with previous estimates obtained via QM/MM MD simulations for pure water as 1.51–2.45 ps^36^ and experimental data indicating that the relaxation time in water is on the order of 1 ps.^100,101^

The CN time correlation functions *C*_CN_(*t*) as obtained using the different NN algorithms are shown in Supporting InformationFigure 9. Since it was again possible to provide an average of all water molecules in the system, the plots show a much higher degree of smoothness. This enables not only the determination of the characteristic relaxation time τ_CN_ via integration but also the calculation of the associated short- and long-time decay constants τ_s_ and τ_l_. The latter can be determined via a double-exponential fit of the form
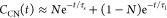
7

**Figure 9:**
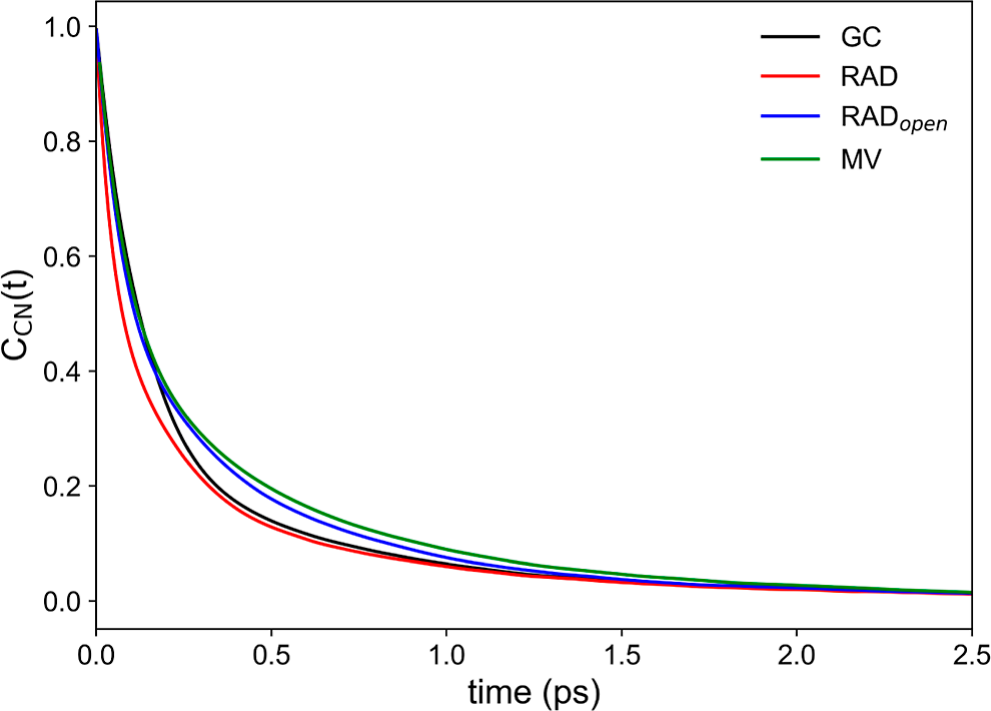
CN autocorrelation functions *C*_CN_(*t*) determined by averaging over 250 water molecules in the system using the GC (black), RAD (red), RAD_open_ (blue), and MV (green) NN algorithm, respectively.

From this, an effective decay constant τ_eff_ can be determined via
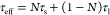
8

The respective data for the relaxation time and the different decay constants are summarized in Supporting Information Table S8. Despite the differences in the algorithms, which result in different decompositions of the first shell ligand assignments, the characteristic parameters determined from the time correlation functions are very similar. In all cases, the relaxation time τ_CN_ of approximately 0.3 ps aligns nicely with the estimates for the effective decay constant τ_eff_. The agreement between the different NN algorithms again shows that the fluctuations in the CNs have similar time correlation properties. This finding supports the explanation of why highly similar estimates for the MRT value are obtained using different algorithms, especially when eliminating short-term fluctuations smaller than the characteristic value for t*. Thus, these data provide another strong argument for the choice of a t*-value of 0.5 ps as this value strikes a balance in being as small as possible while still remaining larger than the characteristic decay constant determined for short-time fluctuation in the CN of pure water. As the τ_CN_ values obtained for the other aqueous systems are on a similar time scale or even faster, the employed t* value represents an adequate reference for water and aqueous solutions as intended in the original derivation of the direct method.^37^

## 4 Conclusions

In this work, several approaches, namely, the GC, RAD, RAD_open_, and MV algorithms, have been combined with the direct method to determine the average CN and the associated MRT of different solvated systems from MD simulation data. With the exception of the GC criterion, all other approaches provide a definition of first shell ligands without any predefined threshold such as a cutoff radius. By evaluating the performance of the different approaches in the characterization of a number of increasingly complex simulation systems, several key observations were made.

Although they are based on an entirely different framework to achieve the first shell ligand assignment, the GC and the RAD-based algorithms result in nearly identical average CNs in good agreement with theoretical and experimental data in the case of the solvated cations Li^+^, Na^+^, and K^+^ in aqueous solution. However, for the investigated anions F^–^, Cl^–^, and Br^–^, the RAD-based methods give average CNs that agree better with theoretical and experimental literature compared to the simpler GC method. On the other hand, the MV algorithm tends to consistently overestimate the CN in all cases.

The shell separation between the first solvation shell and the remaining bulk has been analyzed in terms of the distance of the respective intersection between the two segmented ion-O RDF plots. In addition, the area of overlap of the individual RDFs in the transition region was analyzed. It was found that the RAD method gives the smallest area for all ionic solutes, while at the same time, the distance of intersection aligns best with the minimum in the respective solute–solvent RDFs. This confirms that the RAD approach provides a reliable approach to classify the coordination sphere of solvated ions without the need for any predefined threshold criterion, making it the preferred method for an automated analysis of simulation data in this case.

Also, in the case of the hydrated ions, the number of first shell ligand exchange events that persisted for the minimum excursion time (t* ≥ 0.5 ps) per simulation time *N*^0.5^ was found not to differ significantly for the GC, RAD, and RAD_open_ approaches, although small deviations are observed for the most labile ions K^+^ and Br^–^, respectively. However, in contrast to the differences in the average CNs and the *N*^0.5^ values, the mean residence times τ obtained using the different algorithms proved to be remarkably consistent. It is shown that the MRT is effectively insensitive to the different definitions of the solvation shells, even when the less suitable MV algorithm is used. Only in case of solvated Li^+^, a notably different estimate for the MRT has been obtained based on the MV method when compared with the results obtained by the other algorithms.

These surprising results were further investigated by analyzing the characteristic time frame of short-time fluctuations in the number of ligands *N*^0.0^ and calculating the associated CN autocorrelation functions. The associated relaxation times τ_CN_ were found to be at most 0.3 ps, which implies that short-time fluctuations occur on a shorter time scale as the minimum excursion time t* set to 0.5 ps.

In the case of the more challenging CO_2_ system, which combines cationic and anionic interaction motifs, the MV algorithm produces comparable results in the average CNs compared to the GC method. On the other hand, it was shown that ligands in direct contact with the O atom of the CO_2_ molecule can be excluded from the first solvation shell in the RAD approach. This is a result of the NN definition in the RAD algorithm where, depending on the solute–solvent–solute angle, ligands may be excluded simply because of large differences in the solute–solvent distance compared to other, closer solvent molecules. Thus, the MV algorithm provides a more adequate shell separation of the NNs from a molecular perspective, whereas the RAD approach is suitable for identifying the NNs with respect to the central atom of the investigated solute. Similar to the case of the ionic solutes, the deviations in the average CNs resulting from the four different approaches are not reflected in the determination of the MRT. Again, highly consistent MRT values are obtained for CO_2_ in both aqueous and DCM solution, regardless of the algorithm used to identify first shell ligands.

Also, in this case, the evaluation of the CN autocorrelation points toward a similar relaxation time as observed in the case of the ionic solutes in aqueous solution. Only in the case of CO_2_ in the DCM solution, the question remains whether the t* value of 0.5 ps initially developed for the aqueous case provides an adequate threshold. Although the relaxation times τ_CN_ indeed showed a tendency toward larger values up to 0.5 ps in the GC case, the total number of ligand transitions *N*^0.0^ is notably smaller than in aqueous solution. This can be directly attributed to the higher mass of a CH_2_Cl_2_ molecule, which can be expected to lead to a smaller number of short-time fluctuations.

In the final example, the coordination and ligand exchange dynamics in pure water have been studied based on a simulation at the semiempirical DFTB3/3obwp/D3 level of theory. Similar to the case of ionic solutes, the MV algorithm dramatically overestimates the CN. While the RAD-based approaches show much better agreement, the segmented RDFs again indicate the inclusion of second shell ligands in the first solvation layer. However, also in this example, the determination of the MRT proved to be virtually insensitive to the NN definition. By independently analyzing each water molecule of the simulation system it was shown that the determination of the CN as well as the MRT values resulting from a particular NN algorithm are consistent and only show small standard deviations. The associated CN autocorrelations again point toward a characteristic decay in the ligand fluctuation being approximately 0.3 ps independent of the applied NN algorithm. This provides another strong argument in favor of the applied threshold value of t* = 0.5 ps, providing a suitable balance in eliminating short-time ligand fluctuations on the one hand, while being sufficiently short to discriminate the ligand exchange dynamics of different species (e.g., Li^+^ vs Na^+^ vs K^+^).

Based on the results of this work, the choice in the definition of the first solvation shell to be used depends mainly on the complexity of the studied system and the importance of the associated properties, i.e., whether the properties of the exchange dynamics (CNs or MRT) are of particular interest and to what extent structural properties, such as the formation of a well-separated first solvation shell, are more important for the actual research question.

## Supporting Information Available

Segmented ion-O pair distribution functions for aqueous Li^+^, Na^+^, F^–^, and Cl^–^; integration plots of the overlap area in the segmented pair distribution functions for aqueous Li^+^, Na^+^, K^+^, F^–^, Cl^–^, and Br^–^; example configuration showing RAD ligand assignment for aqueous CO_2_; comparison of the H_2_O–H_2_O pair distribution functions obtained at the DFTB3/3obwp/D3 level of theory with experimental reference plots; density profile obtained for pure water at the DFTB3/3obwp/D3 level of theory; average CNs and MRTs and in pure water obtained using the GC, RAD, RAD_open_, and MV algorithms; Pearson correlation coefficient and Spearman’s rank correlation coefficient for each pair of NN algorithms for aqueous Li^+^, Na^+^, K^+^, F^–^, Cl^–^, and Br^–^; cumulative integration of the overlap area resulting from the segmented ion-O pair distribution functions for aqueous Li^+^, Na^+^, K^+^, F^–^, Cl^–^, and Br^–^; total number of first shell ligand exchange events registered for *t** = 0.0 for aqueous Li^+^, Na^+^, K^+^, F^–^, Cl^–^, and Br^–^; number of first shell ligand exchange events registered for *t** = 0.5 ps per simulation time *N*_ex_^0.5^ and rate coefficient *R*_ex_ of CO_2_ in aqueous and DCM; total number of first shell ligand exchange events registered for *t** = 0.0 ps for both CO_2_ in aqueous and DCM solution and pure water; relaxation time of the autocorrelation function τ_CN_ in ps for aqueous Li^+^, Na^+^, K^+^, F^–^, Cl^–^, and Br^–^; relaxation time of the autocorrelation function τ_CN_ for CO_2_ in H_2_O and DCM; and relaxation time of the autocorrelation function τ_CN_, pre-exponential fitted factor *N*, short-time decay constants τ_s_, long-time decay constants τ_l_, and effective decay constants τ_eff_ determined from the DFTB3 MD simulation of pure water via the GC, RAD, RAD_open_, and MV algorithms (PDF)
