# Supplementary material for: Characterization of the Coordination and Solvation Dynamics of Solvated Systems—Implications for the Analysis of Molecular Interactions in Solutions and Pure H2O
Source: J Chem Theory Comput. 2024 Apr 10;20(8):3028–45. doi: 10.1021/acs.jctc.4c00162 (PMC11044269; doi:10.1021/acs.jctc.4c00162)
Supplement: Supplementary file 1 — ct4c00162_si_001.pdf [file ct4c00162_si_001.pdf]

Characterisation of the Coordination and Solvation Dynamics of Solvated Systems – Implications for the Analysis of Molecular Interactions in Solutions and pure H_2O

Supporting Information

Risnita Vicky Listyarini[a,b], *Bernhard M. Kriesche*[a], *Thomas S. Hofer*[*a]

[a] Institute of General, Inorganic and Theoretical Chemistry
Center for Chemistry and Biomedicine, University of Innsbruck
Innrain 80-82, A-6020 Innsbruck, Austria

[b] Chemistry Education Study Program
Sanata Dharma University, Yogyakarta 55282, Indonesia

[*] Corresponding author Email address: t.hofer@uibk.ac.at (T. S. Hofer)

1 Supporting Information

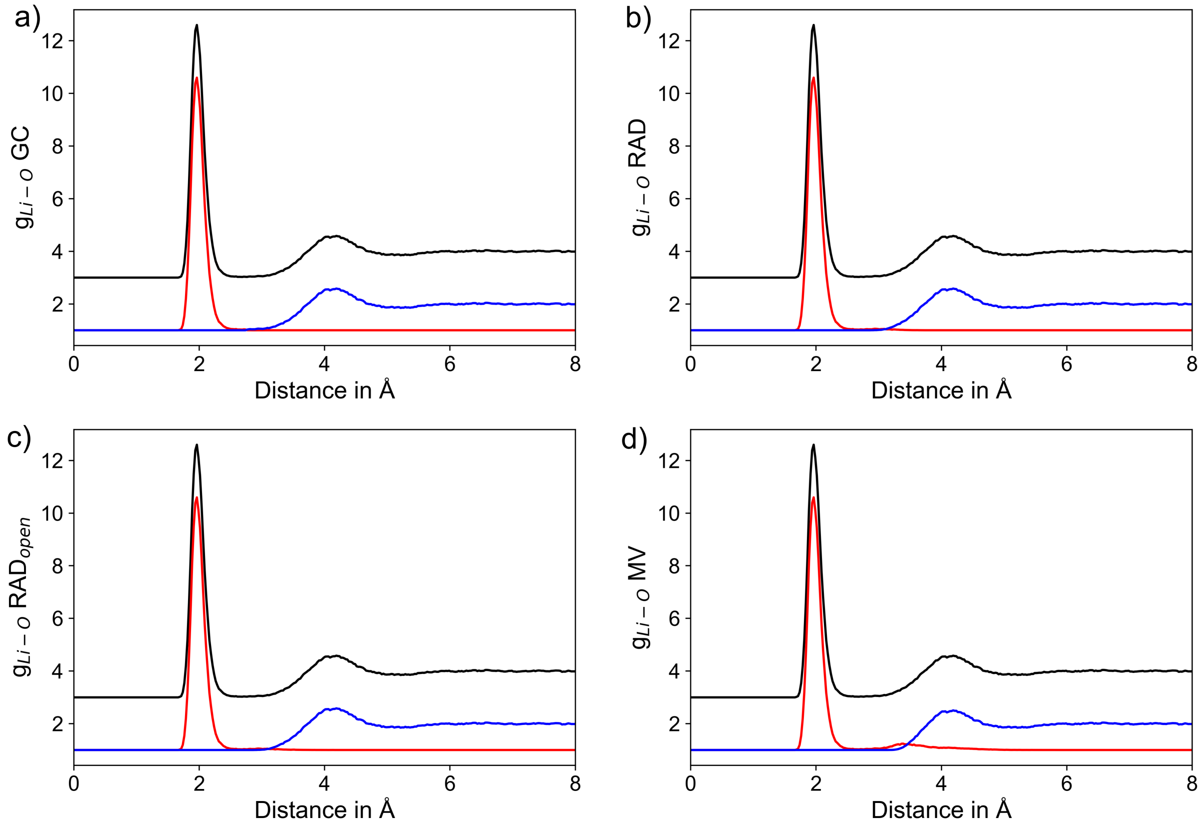

Fig. S1: Radial distribution functions of Li^+-O_{H_2O} pairs considering the entire RDF (black) along with the respective segmentation into the first solvation shell (red) and the remaining solvent (blue) based on the a) GC, b) RAD, c) RAD_{open} and d) MV nearest neighbour algorithms, respectively. To improve the visibility, the total RDF is displayed at an offset of +3.

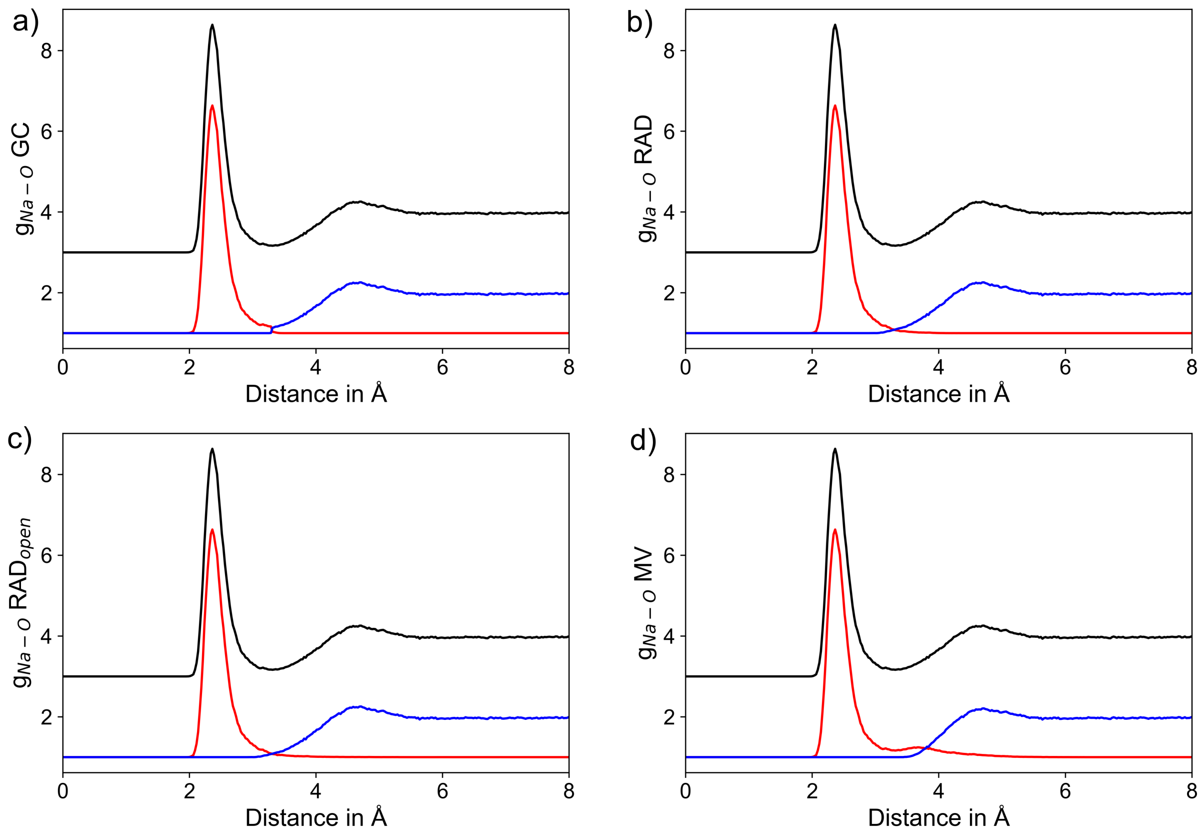

Fig. S2: Radial distribution functions of Na^+-O_{H_2O} pairs considering the entire RDF (black) along with the respective segmentation into the first solvation shell (red) and the remaining solvent (blue) based on the a) GC, b) RAD, c) RAD$_{open}$ and d) MV nearest neighbour algorithms, respectively. To improve the visibility, the total RDF is displayed at an offset of +3.

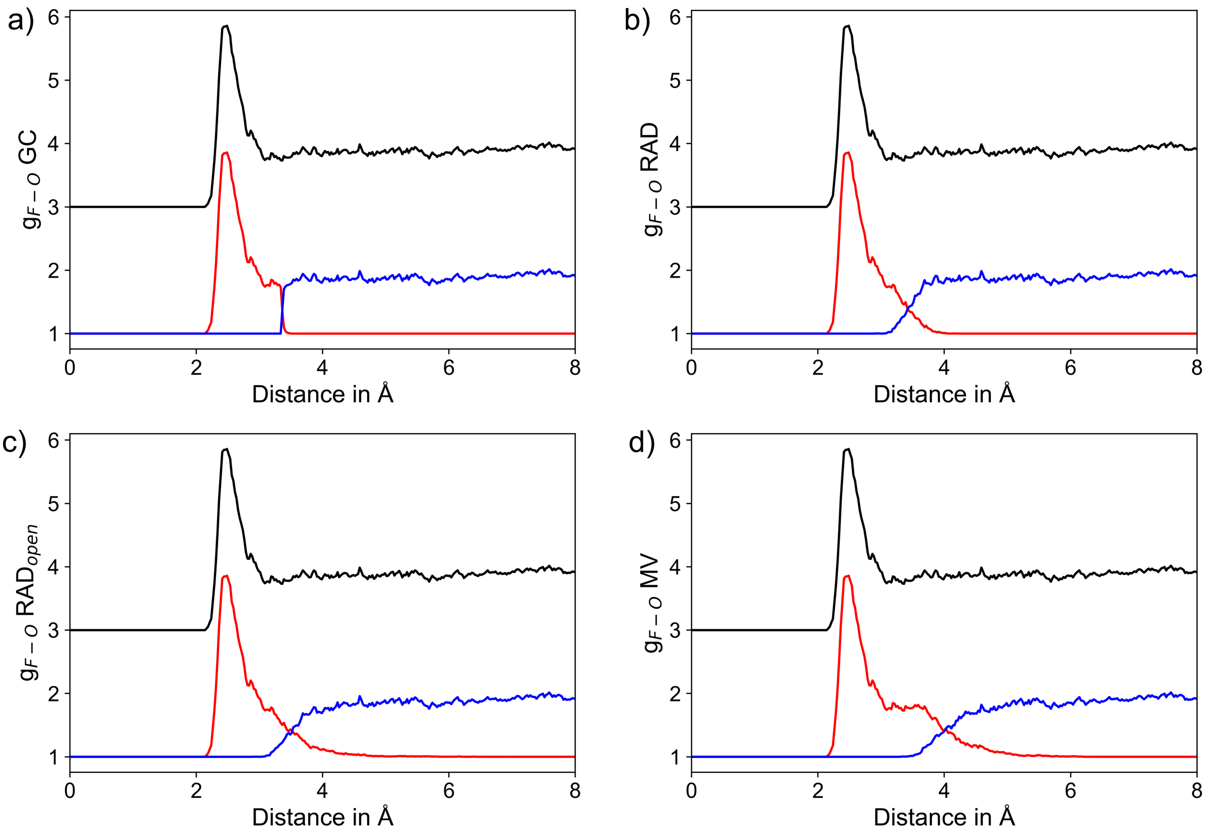

Fig. S3: Radial distribution functions of F^{-}-O_{H_2O} pairs considering the entire RDF (black) along with the respective segmentation into the first solvation shell (red) and the remaining solvent (blue) based on the a) GC, b) RAD, c) RAD_{open} and d) MV nearest neighbour algorithms, respectively. To improve the visibility, the total RDF is displayed at an offset of +3.

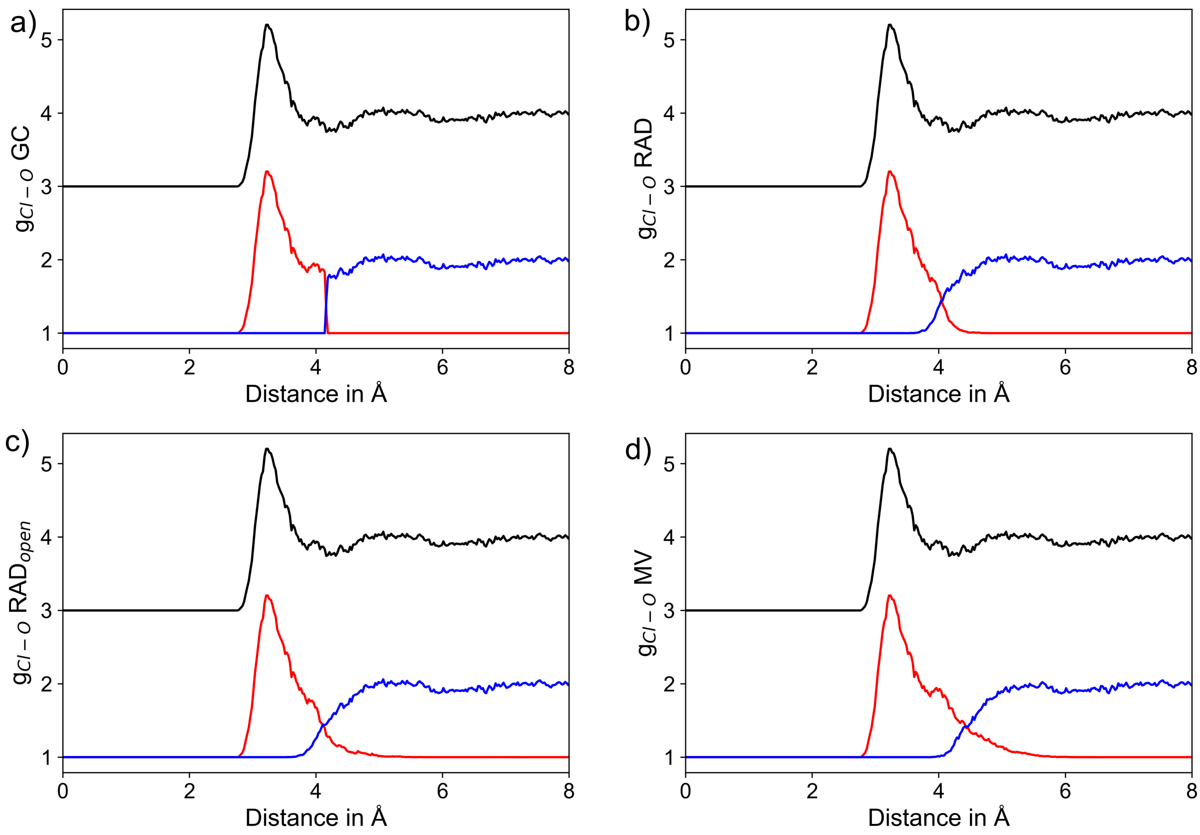

Fig. S4: Radial distribution functions of Cl^{-}-O_{H_2O} pairs considering the entire RDF (black) along with the respective segmentation into the first solvation shell (red) and the remaining solvent (blue) based on the a) GC, b) RAD, c) RAD_{open} and d) MV nearest neighbour algorithms, respectively. To improve the visibility, the total RDF is displayed at an offset of +3.

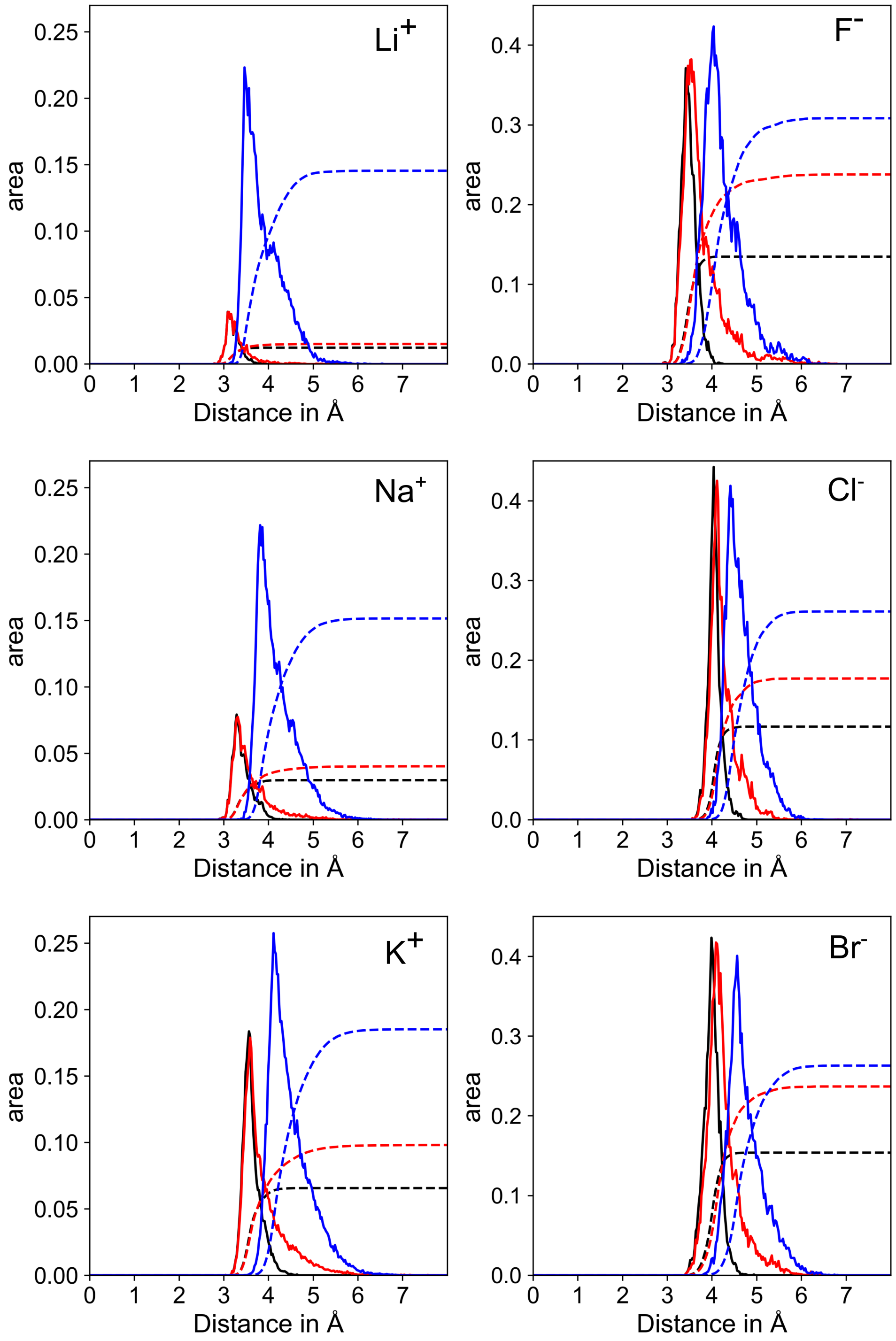

Fig. S5: Overlap area resulting from the segmented ion-O pair distribution functions as well as the cumulative overlap area (dashed lines) determined via trapezoidal integration for aqueous Li^+, Na^+, K^+, F^-, Cl^- and Br^- resulting from the RAD (black), RAD_{open} (red) and MV (blue) nearest neighbour algorithm, respectively.

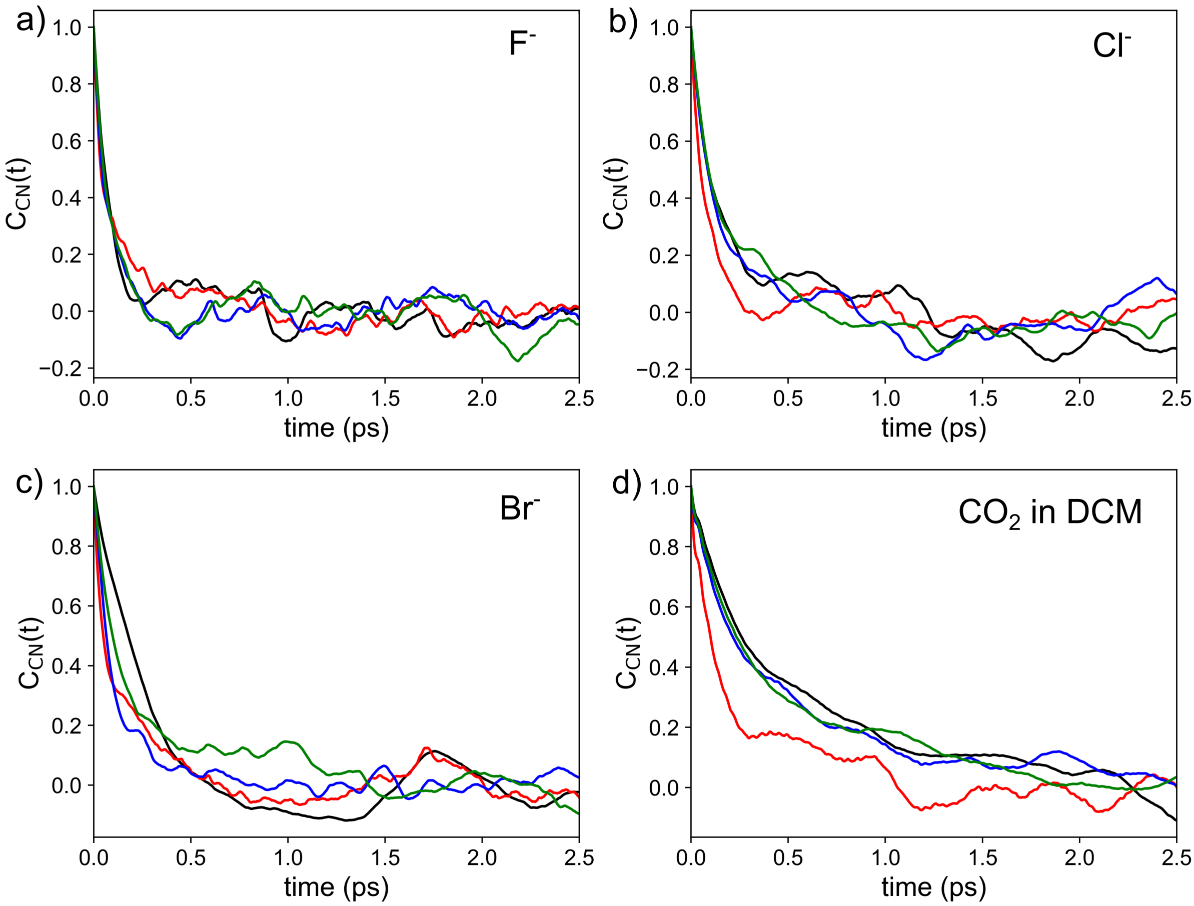

Fig. S6: Coordination number autocorrelation function $C_{\mathrm{CN}}(t)$ determined for a) F^- b) Cl^- c) Br^- in aqueous solution and d) CO_2 in DCM obtained using the GC (black), RAD (red), $\mathrm{RAD_{open}}$ (blue) and MV (green) nearest neighbour algorithm.

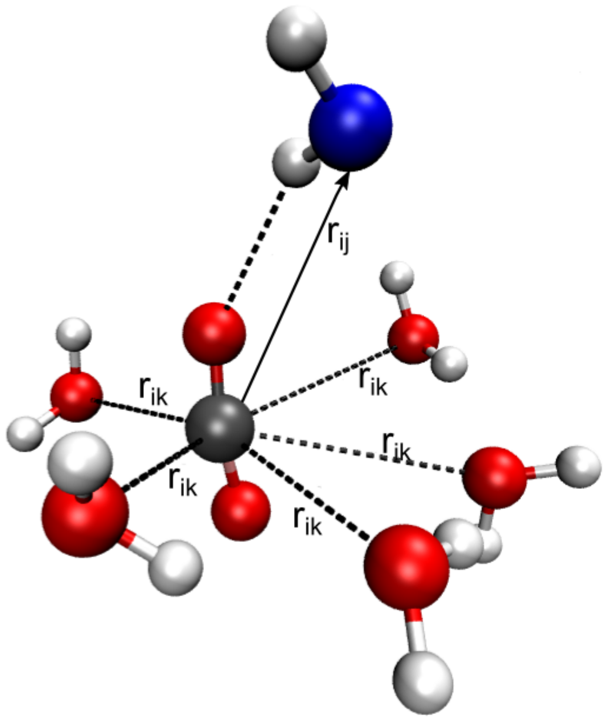

Fig. S7: Example configuration taken from the MD simulation trajectory of aqueous CO_2 with five water molecules interacting with the positively charged central carbon atom *via* charge-dipole interactions shown in red. When considering the water molecule in proximal position of CO_2 (blue) at a distance of r_{ij}, the five closer ligands are considered at distances labelled as r_{ik} according to the RAD approach (see eq. 1 of the main manuscript). Depending on the angle θ_{jik}, the proximal ligand may be excluded from the nearest neighbour assignment in the RAD algorithm due to the large difference between r_{ij}^2 and r_{ik}^2.

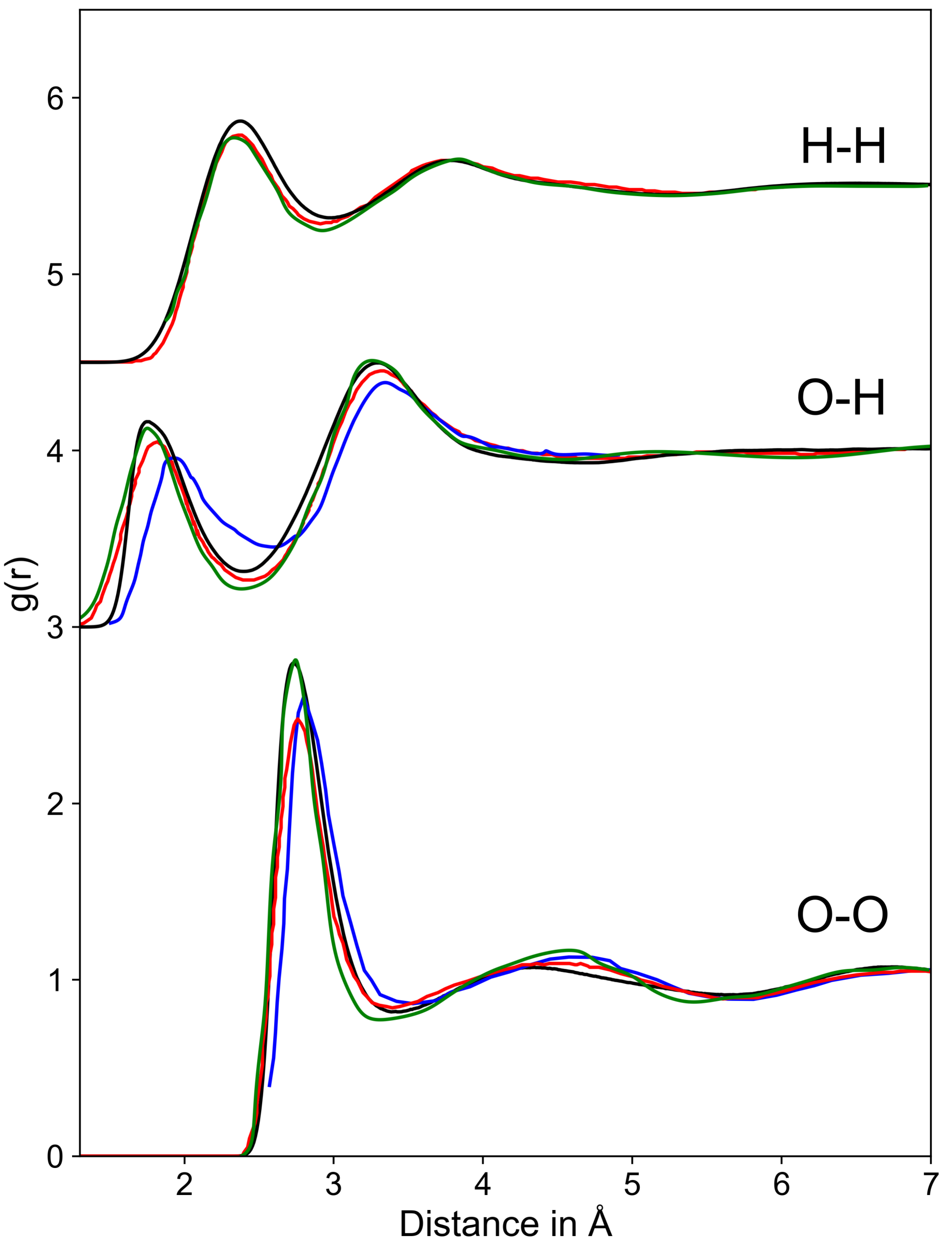

Fig. S8: Radial distribution functions of O-O, O-H, and H-H pairs in pure water treated at DFTB3/3obwp level of theory (black) in comparison with experimental reference data reported by Soper [1] (red), Soper & Benmore [2] (green) and Petterson & Takahashi [3] (blue).

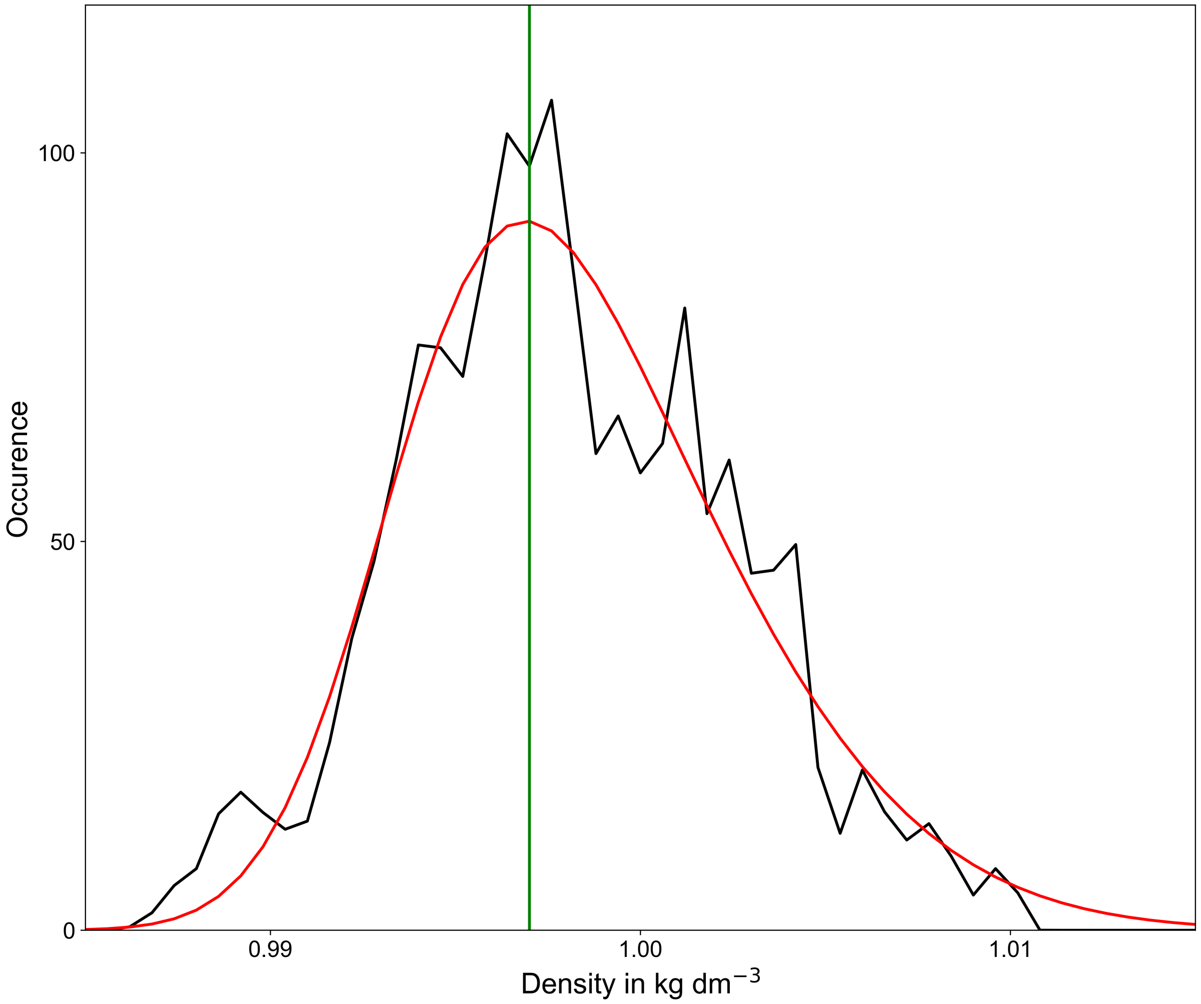

Fig. S9: Density profile of water resulting from the NPT-based MD simulation carried out at the DFTB3/3obwp level of theory (black) and the associated fit to a skewed normal distribution (red). The value for the experimental density 0.997 kg dm^{-3} is shown as the vertical line (green).

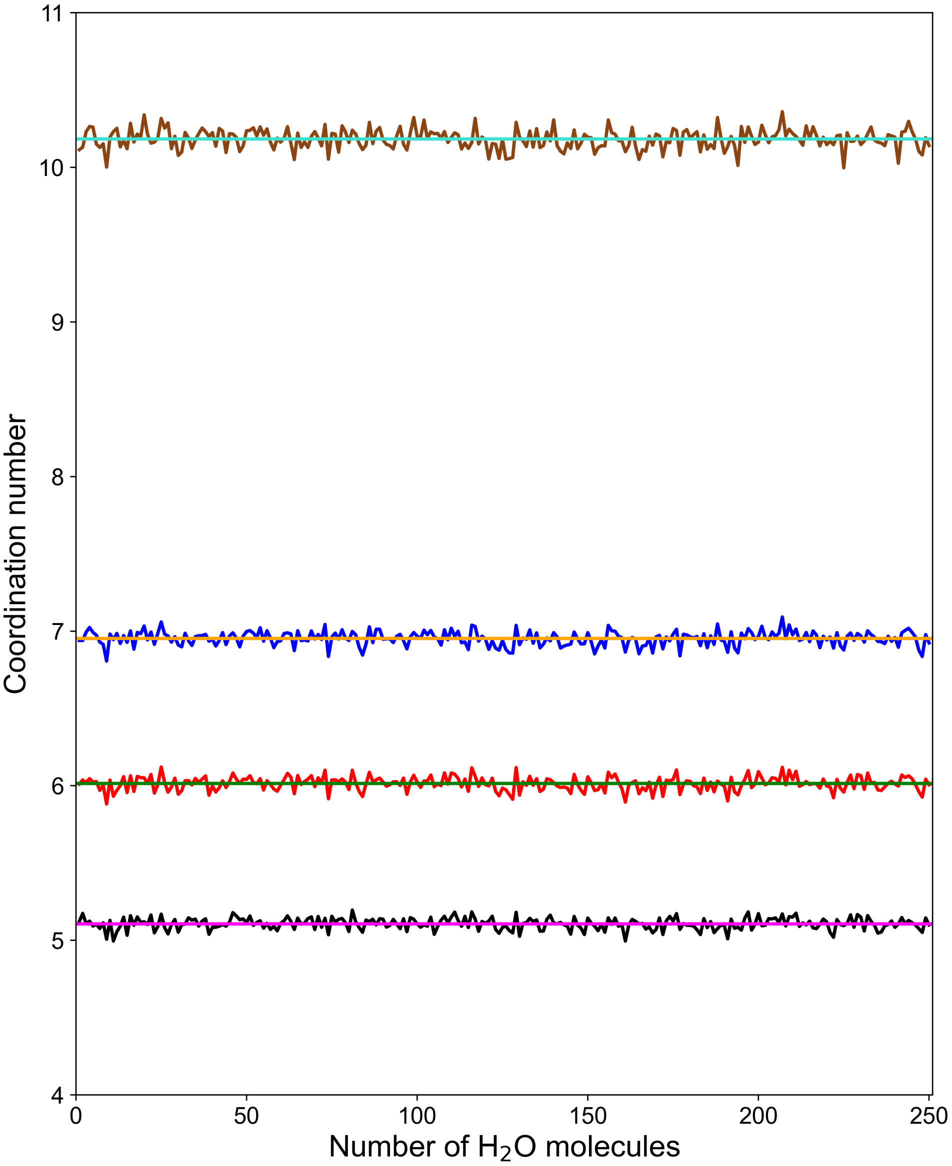

Fig. S10: Average coordination number determined for each of the 250 water molecules in the system based on the GC (black), RAD (red), RAD_{open} (blue) and MV (brown) nearest neighbour algorithm. The respective averages over all water molecules are shown as horizontal lines.

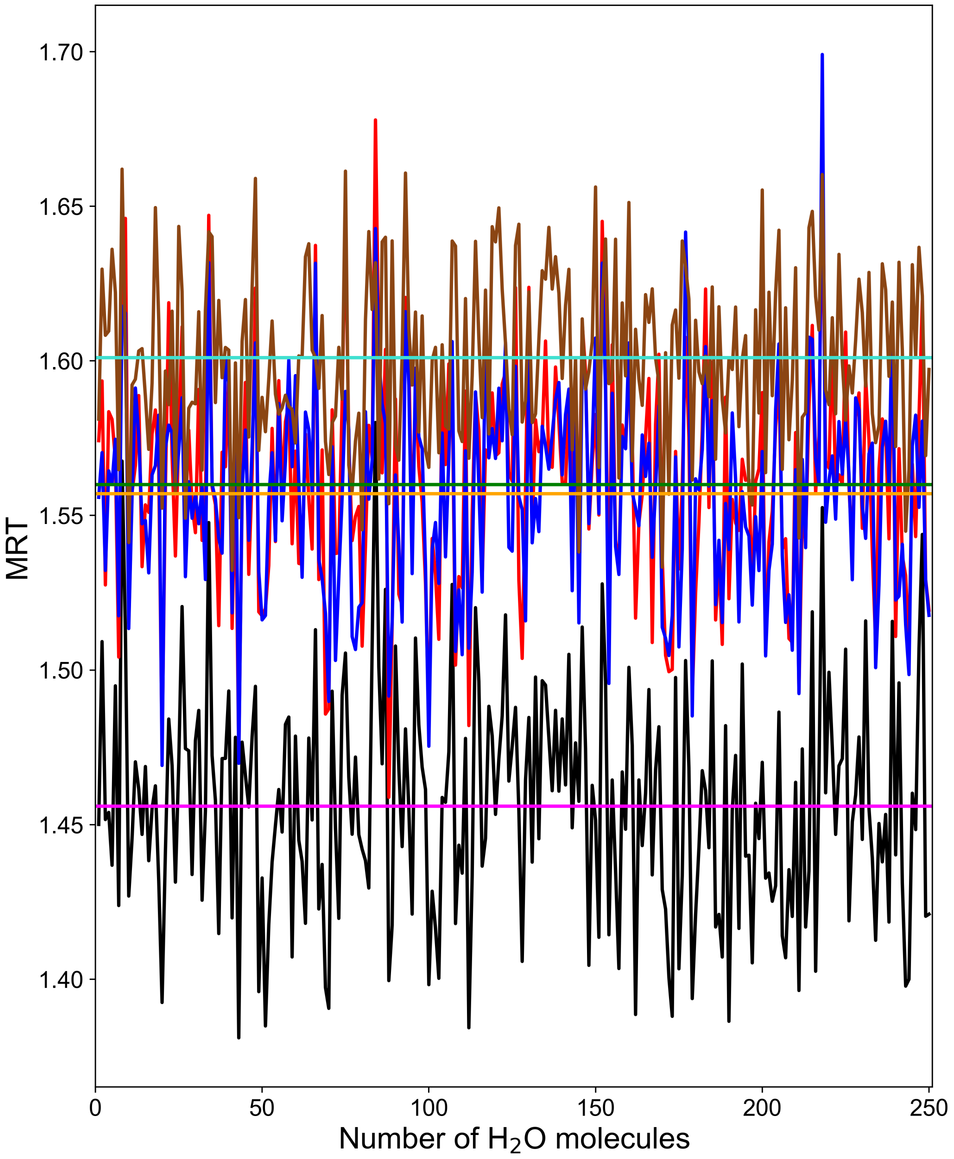

Fig. S11: Mean ligand residence time determined for each of the 250 water molecules in the system based on the GC (black), RAD (red), RAD_{open} (blue) and MV (brown) nearest neighbour algorithm. The respective averages over all water molecules are shown as horizontal lines.

Table S1: Pearson and Spearman rank correlation coefficients determined for different combinations of the GC, RAD, RAD_{open} and MV nearest neighbour algorithms in case of aqueous Li^+, Na^+, K^+, F^-, Cl^- and Br^-, respectively.

Ion	correlation coefficient	GC-RAD	GC-RAD_{open}	GC-MV	RAD-RAD_{open}	RAD-MV	RAD_{open}-MV
Li^+	Pearson	0.575	0.552	0.137	0.943	0.250	0.279
	Spearman	0.574	0.545	0.147	0.945	0.252	0.276
Na^+	Pearson	0.851	0.792	0.243	0.920	0.254	0.335
	Spearman	0.857	0.794	0.238	0.915	0.242	0.322
K^+	Pearson	0.778	0.697	0.331	0.840	0.272	0.468
	Spearman	0.779	0.697	0.334	0.838	0.277	0.456
F^-	Pearson	0.598	0.313	-0.075	0.591	-0.048	0.306
	Spearman	0.591	0.330	-0.070	0.593	0.052	0.285
Cl^-	Pearson	0.461	0.427	0.213	0.657	0.066	0.427
	Spearman	0.469	0.435	0.232	0.653	0.080	0.424
Br^-	Pearson	0.520	0.517	0.330	0.632	0.067	0.458
	Spearman	0.498	0.515	0.343	0.647	0.072	0.433

Table S2: Cumulative area determined via trapezoidal integration of the overlap in the segmented ion–O pair distribution functions for aqueous Li^+, Na^+, K^+, F^-, Cl^- and Br^- resulting from the RAD, RAD_{open} and MV nearest neighbour algorithm, respectively.

Ion	$Area_{RAD}$	$Area_{RAD_{open}}$	$Area_{MV}$
Li^+	0.012	0.015	0.146
Na^+	0.030	0.040	0.151
K^+	0.066	0.098	0.185
F^-	0.135	0.239	0.309
Cl^-	0.117	0.177	0.261
Br^-	0.154	0.237	0.263

Table S3: Total number of first shell ligand exchange events $N_{ex}^{0.0}$ in ps^{-1} for aqueous Li^+, Na^+, K^+, F^-, Cl^- and Br^- registered for $t^* = 0.0$ per simulation time resulting from the GC, RAD, RAD_{open} and MV nearest neighbour algorithm, respectively.

Ion	$N_{GC}^{0.0}$	$N_{RAD}^{0.0}$	$N_{RAD_{open}}^{0.0}$	$N_{MV}^{0.0}$
Li^+	0.68	3.41	4.11	30.85
Na^+	3.97	6.40	7.84	23.38
K^+	10.23	17.02	18.90	29.63
F^-	23.48	36.32	37.42	48.58
Cl^-	22.84	33.75	24.62	37.05
Br^-	29.82	53.51	40.98	45.03

Table S4: Number of first shell ligand exchange events $N_{ex}^{0.5}$ in ps^{-1} registered with minimum excursion time of $t^* = 0.5$ ps per simulation time and the associated rate coefficient R_{ex} determined for CO_2 in aqueous and DCM solution *via* the direct method [4] resulting from the GC, RAD, RAD_{open} and MV nearest neighbour algorithm, respectively.

Solute	Solvent	$N_{GC}^{0.5}$	$N_{RAD}^{0.5}$	$N_{RAD_{open}}^{0.5}$	$N_{MV}^{0.5}$	R_{ex}^{GC}	R_{ex}^{RAD}	$R_{ex}^{RAD_{open}}$	R_{ex}^{MV}
CO_2	H_2O	9.67	4.56	6.22	8.55	4.11	12.34	8.17	5.26
CO_2	DCM	3.47	2.51	2.85	3.43	5.42	14.22	8.55	7.40

Table S5: Total number of first shell ligand exchange events $N_{ex}^{0.0}$ in ps^{-1} for CO_2 in aqueous and DCM solution as well as pure water registered for $t^* = 0.0$ per simulation time resulting from the GC, RAD, RAD_{open} and MV nearest neighbour algorithm, respectively.

Solute	Solvent	$N_{GC}^{0.0}$	$N_{RAD}^{0.0}$	$N_{RAD_{open}}^{0.0}$	$N_{MV}^{0.0}$
CO_2	H_2O	39.76	56.34	50.92	44.96
CO_2	DCM	18.83	35.75	24.38	25.37
H_2O	H_2O	17.36	31.65	30.29	40.67

Table S6: Relaxation time τ_{CN} in ps obtained from the integration of the coordination number autocorrelation function of aqueous Li^+, Na^+, K^+, F^-, Cl^- and Br^- using the GC, RAD, RAD_{open} and MV algorithm, respectively.

	Li^+	Na^+	K^+	F^-	Cl^-	Br^-
GC	0.316	0.351	0.239	0.071	0.081	0.137
RAD	0.213	0.305	0.194	0.081	0.071	0.114
RAD_{open}	0.209	0.293	0.187	0.096	0.131	0.482
MV	0.289	0.217	0.177	0.051	0.075	0.234

Table S7: Relaxation time τ_{CN} in ps obtained from the integration of the coordination number autocorrelation function of CO_2 in H_2O and DCM solution using the GC, RAD, RAD_{open} and MV algorithm, respectively.

	CO_2 in H_2O	CO_2 in DCM
GC	0.270	0.504
RAD	0.100	0.190
RAD_{open}	0.160	0.057
MV	0.181	0.461

Table S8: Relaxation time τ_{CN} in ps obtained from the integration of the coordination number autocorrelation function, pre-exponential fit factor N as well as short-time, long-time and effective decay constants τ_s, τ_l and τ_{eff} determined from the DFTB3 MD simulation of pure water using the GC, RAD, RAD_{open} and MV algorithm, respectively.

	τ_{CN}	N	τ_s	τ_l	τ_{eff}
GC	0.29	0.79	0.13	0.84	0.28
RAD	0.26	0.68	0.070	0.61	0.24
RAD_{open}	0.31	0.60	0.088	0.62	0.30
MV	0.34	0.61	0.097	0.70	0.33

References

(1) Soper, A. K. Joint structure refinement of x-ray and neutron diffraction data on disordered materials: application to liquid water. *J. Phys. Condens. Matter* **2007**, *19*, 335206, DOI: 10.1088/0953-8984/19/33/335206.

(2) Soper, A. K.; Benmore, C. J. Quantum Differences between Heavy and Light Water. *Phys. Rev. Lett.* **2008**, *101*, 065502, DOI: 10.1103/PhysRevLett.101.065502.

(3) Pettersson, L. G.; Takahashi, O. The local structure of water from combining diffraction and X-ray spectroscopy. *J. Non Cryst. Solids: X* **2022**, *14*, 100087, DOI: https://doi.org/10.1016/j.nocx.2022.100087.

(4) Hofer, T. S.; Tran, H. T.; Schwenk, C. F.; Rode, B. M. Characterization of dynamics and reactivities of solvated ions by ab initio simulations. *J Comput Chem* **2004**, *25*, 211–217, DOI: 10.1002/jcc.10374.
